# Allergic disease, corticosteroid use, and risk of Hodgkin lymphoma: A United Kingdom nationwide case-control study

**DOI:** 10.1016/j.jaci.2019.10.033

**Published:** 2020-03

**Authors:** Meena Rafiq, Andrew Hayward, Charlotte Warren-Gash, Spiros Denaxas, Arturo Gonzalez-Izquierdo, Georgios Lyratzopoulos, Sara Thomas

**Affiliations:** a Institute of Health Informatics, UCL, London, United Kingdom; b Institute of Epidemiology and Health Care, UCL, London, United Kingdom; c Faculty of Epidemiology and Population Health, London School of Hygiene and Tropical Medicine, London, United Kingdom

**Keywords:** Allergic disease, Hodgkin lymphoma, corticosteroids, asthma, eczema, allergic rhinitis, risk, atopic dermatitis, aOR, Adjusted odds ratio, CPRD, Clinical Practice Research Datalink, HES, Hospital Episode Statistics, HL, Hodgkin lymphoma, ICD-10, International Classification of Diseases, 10th revision, IM, Infectious mononucleosis, IMD, Index of multiple deprivation, OR, Odds ratio, SES, Socioeconomic status, TYAs, Teenagers and young adults, UK, United Kingdom, UTS, Up to standard

## Abstract

**Background:**

Immunodeficiency syndromes (acquired/congenital/iatrogenic) are known to increase Hodgkin lymphoma (HL) risk, but the effects of allergic immune dysregulation and corticosteroids are poorly understood.

**Objective:**

We sought to assess the risk of HL associated with allergic disease (asthma, eczema, and allergic rhinitis) and corticosteroid use.

**Methods:**

We conducted a case-control study using the United Kingdom Clinical Practice Research Datalink (CPRD) linked to hospital data. Multivariable logistic regression investigated associations between allergic diseases and HL after adjusting for established risk factors. Potential confounding or effect modification by steroid treatment were examined.

**Results:**

One thousand two hundred thirty-six patients with HL were matched to 7416 control subjects. Immunosuppression was associated with 6-fold greater odds of HL (adjusted odds ratio [aOR], 6.18; 95% CI, 3.04-12.57), with minimal change after adjusting for steroids. Any prior allergic disease or eczema alone was associated with 1.4-fold increased odds of HL (aOR, 1.41 [95% CI, 1.24-1.60] and 1.41 [95% CI, 1.20-1.65], respectively). These associations decreased but remained significant after adjustment for steroids (aOR, 1.25 [95% CI, 1.09-1.43] and 1.27 [95% CI, 1.08-1.49], respectively). There was no effect modification by steroid use. Previous steroid treatment was associated with 1.4-fold greater HL odds (aOR, 1.38; 95% CI, 1.20-1.59).

**Conclusions:**

In addition to established risk factors (immunosuppression and infectious mononucleosis), allergic disease and eczema are risk factors for HL. This association is only partially explained by steroids, which are associated with increased HL risk. These findings add to the growing evidence that immune system malfunction after allergic disease or immunosuppression is central to HL development.

## Graphical abstract

**Figure:**
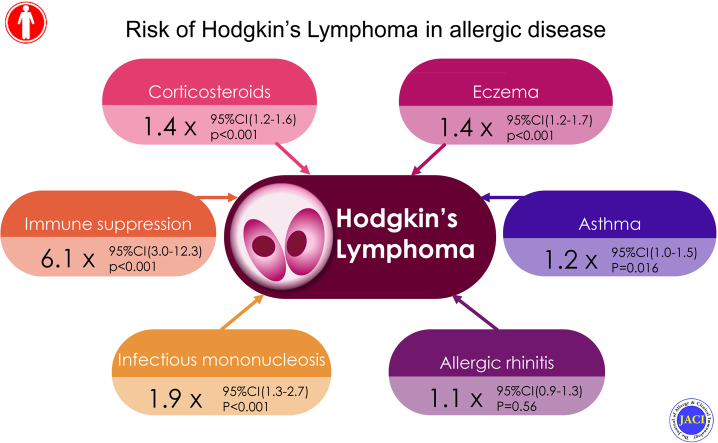

Hodgkin lymphoma (HL) is a cancer of the lymphatic system and is the most common cancer in teenagers and young adults (TYAs) worldwide.^1^^,^^2^ A number of conditions with disordered immune regulation have been associated with an increased risk of HL in TYAs. These include infectious mononucleosis (IM) after EBV infection,^3^, ^4^, ^5^, ^6^, ^7^ HIV infection,^8^, ^9^, ^10^ immunosuppressive therapy,^11^, ^12^, ^13^, ^14^, ^15^, ^16^, ^17^ and several autoimmune diseases, such as multiple sclerosis,^18^ systemic lupus erythematosus,^19^ and rheumatoid arthritis.^20^^,^^21^ Certain HLA genes that are responsible for regulation of the immune system in human subjects have also been associated with increased risk of HL in genetic studies.^22^^,^^23^ These findings together provide support for immune system malfunction playing a central role in the development of HL.

The antigenic stimulation hypothesis has been suggested to explain the underlying mechanism for immune system malfunction in HL development. It proposes that conditions with chronic immune stimulation predispose subjects to hematologic malignancies, such as multiple myeloma, non-HL, and leukemia, by promoting development of randomly occurring pro-oncogenic mutations in actively dividing immune cells.^24^, ^25^, ^26^ There is a growing body of evidence supporting this hypothesis and showing that a number of immune-related cancers, including leukemia, occur as a consequence of immune system malfunction in early life.^27^, ^28^, ^29^

Allergic diseases, including asthma, eczema, and allergic rhinitis, are among the most common perpetrators of chronic immune stimulation. Few studies have investigated the link between allergic diseases and HL, and the results have been conflicting and inconclusive.^24^^,^^25^^,^^30^, ^31^, ^32^, ^33^, ^34^, ^35^ Previous studies have been small scale or relied on small numbers of exposed subjects and therefore might not have had the power to detect associations. No studies have been conducted using electronic health records from primary care, in which allergic disease is predominantly diagnosed and managed, or in the United Kingdom (UK) population, which has one of the highest rates of both HL in TYAs and allergic disease worldwide.^36^^,^^37^

Corticosteroids are a mainstay in the treatment of allergic diseases. Their use is often reserved for more severe cases that have not responded to first-line conventional therapies, and they act primarily through suppression of the immune response. Therefore any association between allergic disease and HL could be intertwined with the effects of steroids: steroid use could modify any effect (because they are a marker of allergic disease severity) or confound it (because they are used in treatment of a range of immune-related diseases that might also be risk factors for HL). Therefore it is important to consider this interplaying role in any study of allergic disease. Some studies have identified steroids as a risk factor for lymphoma,^38^, ^39^, ^40^ although others have found no increased risk^41^^,^^42^; more importantly, it is unclear whether steroid use is an independent risk factor, a marker of allergic disease severity, or a proxy for other immune-related diseases. Furthermore, many of the studies did not differentiate between the types of lymphoma or focused only on topical steroids, adding to the uncertainty surrounding the role of steroid treatment and HL risk.

In this study we used linked primary care electronic health records to determine whether subjects with a history of allergic disease (asthma, eczema, or allergic rhinitis) are at a greater risk of HL in earlier life and whether HL risk varied according to steroid exposure.

## Methods

### Study design and setting

We conducted a matched case-control study using data from the UK Clinical Practice Research Datalink (CPRD), which is linked to Hospital Episode Statistics (HES) inpatient data and index of multiple deprivation (IMD) data. CPRD is an electronic health record database containing prospectively collected anonymized data from UK primary care consultations. It is the largest source of longitudinal primary care data, holding information on 22 million patients, representing approximately 9% of the UK population (in 2013).^43^ Data are available from 1987 onward, when CPRD was first established. It contains information on clinical symptoms, diagnoses (coded using Read codes), investigation results, medications, and referrals to specialists. Practices contributing to CPRD are regularly audited to ensure high data quality and that 95% of prescribing and morbidity events are captured before practices are declared “up to standard” (UTS) for research purposes.^43^

CPRD data used in this study were enhanced by prelinkage to HES. The HES database contains records from every attendance at a National Health Service hospital in England (approximately 125 million episodes per year). Each episode consists of clinical information on diagnoses, procedures, and past medical history coded in International Classification of Diseases, 10th revision (ICD-10). Data are available from April 1997 for patients in practices that have consented to data linkage (54% of all contributing CPRD practices in the UK).^44^

CPRD data were additionally prelinked to information on quintiles of IMD scores in practices that had consented to data linkage. These can be considered to represent a composite ecological (small area–based) measure of the socioeconomic status (SES) of a patient based on the income, employment, disability, educational attainment, and other attributes of the Local Super Output Area of a postcode. The latter typically comprise populations of between 1000 and 3000 residents. All patients had an aggregate IMD score pertaining to the Local Super Output Area in which their general practice is located. For this study, data were extracted from the July 2016 CPRD build and the Set 13 linked data.

### Study population

HL has a bimodal age-specific incidence pattern, with the first peak occurring at between 15 and 34 years of age.^45^ Subjects aged 50 years or less who were actively registered with a CPRD practice that had UTS data between January 1992 and July 2016 were eligible for inclusion in the study. Subjects were excluded if they had an HL diagnosis before entry into the study to avoid inclusion of retrospectively recorded past/prevalent cases, if they had no recorded IMD status, and if they had follow-up of less than 1 year in CPRD.

#### Defining cases with HL

All subjects in the study population with a first diagnosis of HL aged 50 years or less in either CPRD or HES during the study period were included as potential cases (see Table E1 in this article’s Online Repository at www.jacionline.org for Read and ICD-10 code lists). The earliest recorded date of diagnosis was taken as the index date. Cases were excluded if the diagnosis was made within 1 year of registering with a CPRD practice (in accordance with previous studies to ensure that only incident HL diagnoses were identified)^46^ or if there was no event date for the HL diagnosis.

#### Defining matched control subjects

Six control subjects for each case were selected using individual matching on age at index date (±1 year), sex, and duration of active follow-up time (±2 years). A matched design was an efficient way to deal with the potential contributing effects of these variables. Concurrent sampling was used to match HL cases to control subjects who were HL free at the index date of the case while being under active follow-up in a UTS CPRD practice with a similar length of follow-up time before the index date. These subjects could not have an HL diagnosis at the time of matching (index date) but could go on to have HL in the future. This method allowed “matching on time” with cases in this dynamic population.^47^ Each control subject was assigned an index date corresponding to the date of diagnosis of their matched case.

#### Defining patients with allergic disease

A diagnosis of allergic disease was defined as a coded diagnosis of asthma, eczema, or allergic rhinitis in CPRD or HES at any point before the index date. Because we are interested in both incident and prevalent cases of allergic disease, subjects with a diagnosis at any point in their medical record before the index date were classed as having an allergic disease (see Table E2 in this article’s Online Repository at www.jacionline.org for Read and ICD-10 code lists). The total number of allergic diseases (with a maximum of 3) and the date and age of first reporting of allergic disease diagnosis were recorded (categorized as infant [<1 year], child [1-17 years], or adult [≥18 years] onset).

#### Defining corticosteroid use

Corticosteroid use was defined as coded use of any corticosteroid (subclassified as inhaled, topical, oral or intravenous/intramuscular) in CPRD at any point more than 6 months before the index date (see Table E3 in this article’s Online Repository at www.jacionline.org for the code list). A “lag time” of 6 months before the index date was used in line with previous studies to reduce the possibility of reverse causality in the months immediately before HL diagnosis because early symptoms of undiagnosed HL might lead to steroid treatment in the period leading up to the diagnosis.^48^ Steroid use was further classified by the frequency of use during follow-up (total number of coded issues before 6 months before index date).

#### Covariates and mediators

**Fig 1:**
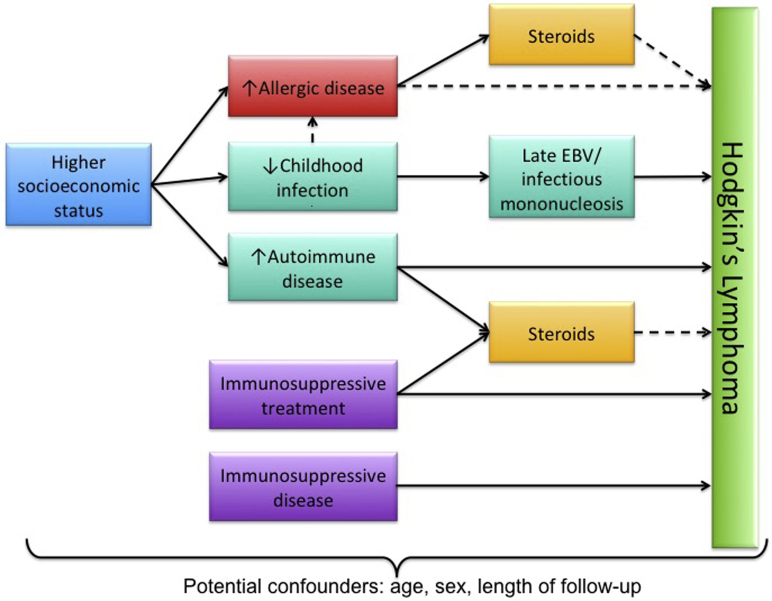
Directed acyclic graph for the study. *Solid lines* indicate assumed associations from previous studies, and *dashed lines* indicate proposed associations examined in the current analysis.

We used a directed acyclic graph to inform the identification of potential covariates and mediators and to avoid collider bias (Fig 1). Covariates included the matched variables of age, sex, and follow-up time and SES (by using quintiles of 2010 IMD). A prior diagnosis of IM or immunosuppressive conditions were also included based on a recorded diagnosis in HES or CPRD before the index date because these are established risk factors for HL. For IM, codes for EBV infection, positive laboratory test results, and IM caused by other viruses were included (see Table E4 in this article’s Online Repository at www.jacionline.org). When classifying immunosuppression, congenital, acquired, and iatrogenic causes were included (see Table E5 in this article’s Online Repository at www.jacionline.org for code lists).

### Statistical analysis

#### Primary analyses

We initially described the baseline characteristics of cases and control subjects. Univariable conditional logistic regression (matched on age at index date, sex, and follow-up duration) was used to generate odds ratios (ORs) for the association between each of the exposure variables and HL, followed by multivariable conditional logistic regression adjusting for all other variables in the model. Interaction terms were subsequently introduced to investigate potential effect modification of the association between HL incidence and allergic disease by age, sex, and SES.

A further analysis was conducted on the final regression model, categorizing allergic disease as a linear rather than binary variable to take into account the number of allergic diagnoses. We assessed for linear trend by number of allergic diagnoses, first by estimating the linear effect using likelihood ratio tests and then by investigating departure from linearity by comparing models in which allergic disease was added as a nonlinear versus a linear term. We used 95% CIs and an implied 5% level of statistical significance to minimize the risk of a type 1 error.

We repeated the analyses with alternative exposure definitions in which each allergic disease was considered separately. First, we constructed a cross-tabulation comparing the frequency of combinations of allergic diseases in cases and control subjects. Then we repeated the conditional logistic regression analysis described above, with asthma, eczema, and allergic rhinitis included as separate variables to evaluate their independent effect on HL incidence after adjusting for each other and other variables in the model. Interaction terms were introduced to investigate for potential effect modification of the estimated risk associated with each allergic disease by age, sex, and SES strata and also other allergic disease. In supplementary analyses, for each of the 3 allergic diseases separately, using likelihood ratio tests, we examined whether a model in which they were categorized as infant/childhood/adult onset differed from a model in which they were considered as yes-no variables independent of age of onset. Where there was evidence for heterogeneity, stratum-specific adjusted odds ratios (aORs) were estimated.

#### Secondary analyses

A secondary analysis was conducted incorporating steroid use into the final model to assess for potential effect modification when stratifying by steroid use and to investigate the extent to which the effect of variables might be confounded by steroid treatment by comparing effect estimates before and after adjustment for steroid use. The effect of steroids was also assessed before and after adjustment for other variables, both collectively (any steroid use) and stratified by route of administration (inhaled, topical, oral, or intravenous/intramuscular). We assessed for a potential dose-response relationship by estimating the linear effect of number of steroid prescriptions before the index date on HL risk and by route of administration (ordered according to strength/level of systemic absorption) using likelihood ratio tests, as described above.

#### Sensitivity analysis

A sensitivity analysis was performed restricted to subjects with HES-linked data, and effect estimates were compared with estimates of the whole case-control population. Analyses were performed with Stata software (version 15; StataCorp, College Station, Tex).

### Ethics approval and consent to participate

The protocol for this project was approved by the London School of Hygiene and Tropical Medicine Ethics Committee (reference 11182) and the Independent Scientific Advisory Committee for MHRA Database Research (protocol no. 16_237). Generic ethical approval for observational studies conducted with anonymized CPRD data with approval from Independent Scientific Advisory Committee has been granted from a National Research Ethics Service Committee. The study was performed in accordance with the Declaration of Helsinki.

## Results

There were 1236 incident cases of HL in this study individually matched to 7416 control subjects. Table I shows the baseline characteristics of subjects in the case-control sample. Mean follow-up time was 6 years. Cases were more likely to be immunosuppressed (1% vs 0.2%), have a history of IM (4% vs 2%), and have a diagnosis of at least 1 of the 3 allergic diseases (41% vs 33%, Table I). Treatment with steroids was more commonly seen in cases than in control subjects for all routes of administration, with significantly more cases having 2 or more steroid prescriptions during follow-up when compared with control subjects (43% vs 34%, *P* < .001; Table I). Cross-tabulation of combinations of allergic diseases showed increased prevalence of asthma (19% vs 15%), eczema (21% vs 16%), and asthma and eczema combined (7% vs 4%) in cases compared with control subjects (Table II). Distribution of all other exposure variables did not differ substantially between cases and control subjects (Table I).

**Table I:** Baseline characteristics of patients with HL and control subjects

Characteristics	Patients with HL (n = 1236)	Control subjects (n = 7416)	*P* value∗
Mean years of follow-up† (SD [range])	6.03 (5.00 [0.01-26.42])	6.01 (4.96 [0.00-26.87])	.9
Male sex†	702 (56.8%)	4212 (56.8%)	1.00
Age at start of follow-up (y)			.98
0-10	208 (16.8%)	1265 (17.1%)	
11-20	241 (19.5%)	1396 (18.8%)	
21-30	357 (28.9%)	2129 (28.7%)	
31-40	331 (26.8%)	2026 (27.3%)	
41-50	99 (8.0%)	600 (8.1%)	
Age at index date† (y)			1.00
0-10	35 (2.8%)	210 (2.8%)	
11-20	239 (19.3%)	1434 (19.3%)	
21-30	337 (27.3%)	2002 (27.3%)	
31-40	355 (28.7%)	2129 (28.7%)	
41-50	270 (21.8%)	1621 (21.9%)	
IMD quintile			.09
5 (most deprived)	251 (20.3%)	1598 (21.6%)	
4	277 (22.4%)	1641 (22.1%)	
3	246 (19.9%)	1442 (19.4%)	
2	221 (17.9%)	1325 (17.9%)	
1 (least deprived)	241 (19.5%)	1410 (19.0%)	
Immunosuppression	16 (1.3%)	15 (0.2%)	<.001
IM	43 (3.5%)	131 (1.8%)	<.001
Allergic disease‡	500 (40.5%)	2429 (32.8%)	<.001
Steroid use	731 (59.1%)	3714 (50.1%)	<.001
Inhaled	294 (23.8%)	1548 (20.9%)	.02
Topical	604 (48.9%)	3011 (40.6%)	<.001
Oral	110 (8.9%)	449 (6.1%)	<.001
Intravascular/intramuscular	30 (2.4%)	112 (1.5%)	.02
No. of steroids			<.001
0	505 (40.9%)	3702 (49.9%)	
1	201 (16.3%)	1178 (15.9%)	
≥2	530 (42.9%)	2536 (34.2%)	
Median no. of steroids (IQR)	1 (0-4)	1 (0-3)	<.001

*IQR*, Interquartile range.

∗ *P* value from χ^2^ test or Mann-Whitney *U* test for continuous variables.

† Matched variables.

‡ Defined as diagnosis of asthma and/or eczema and/or allergic rhinitis during the follow-up period.

**Table II:** Frequency of concurrent allergic diseases: Proportion of cases and control subjects with a diagnosis of 1 or more allergic conditions

	Asthma	Eczema	Allergic rhinitis	All 3
Concurrent allergic diagnoses in cases (n = 1236)				
Asthma	18.9% (233)	6.6%(82)	4.6% (57)	
Eczema	6.6% (82)	21.0% (260)	4.4% (55)	
Hay fever	4.6% (57)	4.4%(55)	13.9% (172)	
All 3				2.4% (29)
Concurrent allergic diagnoses in control subjects (n = 7416)				
Asthma	15.2% (1129)	4.4% (323)	4.2% (308)	
Eczema	4.4% (323)	15.6% (1154)	3.2% (241)	
Hay fever	4.2% (308)	3.2% (241)	12.4% (918)	
All 3				1.4% (100)
*P* value∗	.001	<.001	.13	

∗ *P* value for χ^2^ test comparing allergic disease in cases and control subjects.

### Immunosuppression

Immunosuppression was by far the strongest risk factor for HL incidence in this study. Immunosuppressed subjects had 6 times greater odds of HL on univariable analysis (*P* < .001). There was very little change in OR after adjusting for other variables, indicating the effect was independent of SES, allergic disease, and IM (aOR, 6.18; 95% CI, 3.04-12.57; *P* < .001). A slight attenuation in OR was noted after adjusting for steroid use (aOR, 6.05; 95% CI, 2.97-12.33; *P* < .001), indicating that part of the effect of immunosuppression on HL risk might be attributable to steroid use (Table III).

**Table III:** Association between exposures and HL incidence (≤50 years)

Variable	Univariable OR (95% CI)	aOR∗ (95% CI)	OR after adjustment for steroids (95% CI)
Immunosuppression	6.36 (3.15-12.87)	6.18 (3.04-12.57)	6.05 (2.97-12.33)
*P* value	<.001	<.001	<.001
IM	2.00 (1.41-2.84)	1.89 (1.33-2.68)	1.87 (1.31-2.67)
*P* value	<.001	<.001	.001
Allergic disease	1.42 (1.25-1.62)	1.41 (1.24-1.60)	1.25 (1.09-1.43)
*P* value	<.001	<.001	.002
Deprivation quintile			
5 (most deprived)	Reference	Reference	Reference
4	1.08 (0.89-1.29)	1.09 (0.90-1.30)	1.08 (0.90-1.30)
3	1.09 (0.90-1.32)	1.08 (0.89-1.31)	1.07 (0.89-1.30)
2	1.06 (0.87-1.29)	1.05 (0.86-1.28)	1.04 (0.86-1.27)
1 (least deprived)	1.09 (0.90-1.32)	1.06 (0.88-1.29)	1.05 (0.87-1.28)
*P* value	.47†	.69†	.75†
Steroid use	1.51 (1.33-1.72)	1.38 (1.20-1.59)	—
*P* value	<.001	<.001	—

∗ Matched on age, sex, and follow-up time and adjusted for other variables in the model (region, deprivation, immunosuppression, atopy, and IM); *P* values were from the likelihood ratio test.

† *P* value for test for linear trend.

### IM

IM was associated with double the odds of HL on univariable analysis (*P* < .001). There was minimal attenuation of the effect in mediation models after adjusting for immunosuppression, SES, and allergic disease (aOR, 1.89; 95% CI, 1.33-2.68; *P* < .001) and negligible change in the OR after adjusting for steroid use (Table III). This indicates the effect of IM on HL is independent of these variables.

### Allergic disease

A previous diagnosis of 1 or more allergic diseases was associated with 1.4-fold greater odds of HL (*P* < .001), with minimal change after adjusting for other variables (aOR, 1.41; 95% CI, 1.24-1.60; *P* < .001; Table III). The risk of HL increased with increasing numbers of allergic diagnoses (*P* for linear trend < .001, Table IV). When analyzing by specific allergic disease type, eczema and asthma were associated with increased risk of HL (aOR 1.41 [95% CI, 1.20-1.65; *P* < .001] and 1.23 [95% CI, 1.04-1.45; *P* = .016], respectively) with no evidence of an association between allergic rhinitis and HL (Table IV).

**Table IV:** Association between atopic diseases and HL incidence (<50 years)

Variable	Univariable OR	aOR∗	OR after adjustment for steroids
Asthma	1.31 (1.11-1.53)	1.23 (1.04-1.45)	1.15 (0.97-1.36)
*P* value	.001	.016	.11
Eczema	1.47 (1.26-1.72)	1.41 (1.20-1.65)	1.27 (1.08-1.49)
*P* value	<.001	<.001	.005
Allergic rhinitis	1.15 (0.96-1.37)	1.06 (0.88-1.27)	0.99 (0.83-1.19)
*P* value	.13	.56	.94
Immunosuppression	6.36 (3.15-12.87)	6.05 (2.98-12.30)	5.94 (2.91-12.10)
*P* value	<.001	<.001	<.001
IM	2.00 (1.41-2.84)	1.88 (1.32-2.68)	1.87 (1.31-2.66)
*P* value	<.001	<.001	.001
Steroid use	1.51 (1.33-1.72)	1.39 (1.21-1.60)	—
*P* value	<.001	<.001	—
Topical steroid	1.46 (1.28-1.66)	1.34 (1.17-1.54)	—
*P* value	<.001	<.001	—
Inhaled steroid	1.20 (1.03-1.39)	1.03 (0.87-1.23)	—
*P* value	.017	.73	—
Oral steroid	1.54 (1.23-1.92)	1.30 (1.02-1.65)	—
*P* value	<.001	.036	—
Intravascular/intramuscular steroid	1.63 (1.08-2.46)	1.55 (1.03-2.35)	—
*P* value	.019	.037	—
No. of steroids	1.01 (1.00-1.01)	1.00 (1.00-1.01)	—
*P* value	.003	.37	—
No. of atopic diseases			
0	Reference	Reference	Reference
1	1.43 (1.24-1.64)	1.42 (1.23-1.63)	1.27 (1.09-1.47)
2	1.30 (1.04-1.63)	1.27 (1.01-1.60)	1.10 (0.87-1.39)
3	2.05 (1.34-3.13)	2.04 (1.34-3.13)	1.75 (1.14-2.68)
*P* value†	<.001	<.001	.005

*P* values were from the likelihood ratio test.

∗ Matched on age, sex, and follow-up time and adjusted for other variables in the model (SES, immunosuppression, atopic diseases, and IM).

† *P* value for test for linear trend.

In a supplementary analysis comparing age of allergic disease onset, asthma and allergic rhinitis had similar average age of onset in cases and control subjects. However, for eczema, the median age of onset was 15 years in control subjects and 20 years in cases (*P* = .004). Relatedly, there were significantly more incidences of adult-onset eczema among cases than control subjects (54% vs 44%, see Table E6 in this article’s Online Repository at www.jacionline.org), with strong evidence that the effect of eczema on HL risk differed according to age of eczema onset (*P* = .006). Only adult-onset eczema was associated with increased odds of HL (aOR, 1.73; 95% CI, 1.40-2.13; *P* < .001; see Table E7 in this article’s Online Repository at www.jacionline.org). There was no evidence of heterogeneity of effect estimates by age of onset for asthma or allergic rhinitis (*P* = .33 and .27, respectively; data not shown).

In the secondary analysis, after adjusting for steroid use, associations between allergic disease and eczema with HL were attenuated but still found to be significant (aOR, 1.25 [95% CI, 1.09-1.43; *P* = .002] and 1.27 [95% CI, 1.08-1.49; *P* = .005], respectively; Table III, Table IV). In asthmatic patients, after adjustment for steroids, there was no increased risk of HL (Table III, Table IV). There was no difference in effect estimates when stratifying by steroid use (Table III, Table IV), and there was no evidence of effect modification by age at index date, sex, or SES (test for interaction *P* = .12, .063, and .41, respectively; additional analyses not shown in tables).

**Table V:** Association between allergic diseases and HL incidence stratified by steroid use

Variable	Used steroid, aOR∗ (95% CI)	Never used steroids, aOR∗ (95% CI)	*P* value for effect modification
Allergic disease	1.17 (0.99-1.37)	1.48 (1.15-1.90)	
*P* value	.064	.002	.12
Asthma	1.00 (0.83-1.21)	1.85 (1.34-2.56)	
*P* value	.99	<.001	.002
Eczema	1.27 (1.07-1.51)	1.27 (0.81-1.99)	
*P* value	.007	.31	.99
Allergic rhinitis	0.96 (0.78-1.18)	1.14 (0.77-1.71)	
*P* value	.69	.51	.45
Immunosuppression	9.08 (3.59-22.96)	2.67 (0.71-10.10)	
*P* value	<.001	.15	.12
IM	1.82 (1.17-2.83)	1.96 (1.08-3.56)	
*P* value	.008	.028	.85

*P* values were from the likelihood ratio test.

∗ Matched on age, sex, and follow-up time and adjusted for other variables in the model (SES, immunosuppression, atopic diseases, and IM).

### Corticosteroid use

Previous steroid use for any indication was associated with increased risk of HL. Subjects with a history of steroid use at any time before 6 months before the index date had 1.5-fold increased odds of HL (OR, 1.51; 95% CI, 1.33-1.72; *P* < .001). All routes of administration were associated with increased risk, with the strongest associations seen for intravenous/intramuscular administration, followed by oral, topical, and then inhaled steroids (Table III, Table IV). After adjusting for other variables, including allergic disease and other immune conditions, steroid use remained a significant risk factor for HL development (aOR, 1.38; 95% CI, 1.20-1.59; *P* < .001), and this was seen for all routes of administration except inhaled steroids (Table III, Table IV).

### Sensitivity analysis

Restricting the analysis to patients who had HES-linked data available (59.6% of all patients in this study) produced similar effect estimates for variables across all regression analyses.

## Discussion

This study shows that allergic disease and steroid use for any indication are associated with an increased risk of HL before the age of 50 years. A previous diagnosis of eczema, but not asthma or allergic rhinitis, is associated with development of HL, with this effect being concentrated in patients with adult-onset eczema. This effect does not differ by steroid exposure and persists after adjustment for steroid use. Previously established risk factors for HL involving immune dysfunction were also found in this study to be important risk factors for HL in early life. Immunosuppressed subjects had a 6-fold increased odds of HL, and those with a history of IM had almost double the odds.

### Comparison with the literature

Associations between allergic conditions and HL have been inconsistent and inconclusive in the literature (see Table E8 in this article’s Online Repository at www.jacionline.org). Söderberg et al^25^ conducted a Swedish population-based case-control study of 2394 patients with HL that found asthma was associated with a 40% reduced risk of HL. This study relied on hospital discharge summary data, which are likely to include only severe asthma, and results were based on only 18 exposed cases.

Vineis et al^30^ conducted an Italian population-based case-control study that reported a 50% reduced risk of HL in patients with allergic rhinitis but no effect of asthma or eczema. This was a small study of 354 cases and relied on face-to-face interviews of adults, which might introduce recall bias of childhood exposures.

Cozen et al^31^ carried out a twin study comparing 188 HL-discordant twin pairs in the United States using questionnaires. They found that eczema was associated with a 4-fold increased risk of HL but was based on only 19 discordant pairs for the exposure.

A number of further studies have concluded no association between allergic disease and HL risk.^24^^,^^32^, ^33^, ^34^, ^35^ These were small-scale case-control studies of up to 585 cases and relied on retrospectively collected exposure data from telephone interviews and questionnaires. Misclassification is therefore likely because of exposures being self-reported. Additionally, many of the studies included a diagnosis of HL at any age, which could produce misleading results because studies have shown HL in subjects aged less than 50 and more than 50 years are likely to have different causes and might even be 2 separate disease entities.^49^, ^50^, ^51^

Existing studies on steroid use and HL are also limited and have produced conflicting findings. One study found an increased risk of any lymphoma with oral steroid use but no increased risk with topical steroids after adjusting for other factors.^39^ A second study focusing specifically on HL found no increased risk, even at considerable and cumulative doses of oral steroids; however, this study focused on HL cases aged more than 50 years.^41^ Some further studies of topical steroid use have shown increased HL risk in a dose-response fashion, with increasing duration of exposure and potency,^38^^,^^40^ but others have shown no increased risk, even with moderate/highly potent topical steroids.^42^

### Strengths and limitations

We know of no previous studies assessing the association between allergic diseases with HL using prospectively collected population-based primary care electronic health records data and considering the potential interplay with steroid treatment. CPRD data are representative of the UK population across a number of demographic variables,^43^ which supports the external validity of the findings. Allergic conditions are predominantly diagnosed and treated in primary care, making general practitioner electronic health records an ideal setting for examining them. Recording of asthma diagnosis in CPRD has high validity against gold standard diagnosis, with a positive predictive value of 86.4%.^52^ HL diagnoses have high validity in CPRD when compared with gold standard national cancer registration data (positive predictive value for lymphoma, 89.6%; sensitivity, 97.3%).^53^ Combined use of primary and secondary care HES-linked data further improved the validity of exposures and outcomes by supplementing general practitioner records with hospital data to improve capture of diagnoses. We used detailed exploration of diagnostic codes verified by 2 clinicians and crosschecking with existing code lists in the literature to further improve diagnostic accuracy. Rates of allergic diseases and HL in the study population showed a similar distribution to that reported in the literature. The large study sample enabled the precise estimation of associations, providing adequate power to identify associations when the effect size is small. Prospectively collected data have low risk of recall bias, unlike other types of data used in previous studies.

As for all observational studies based on routine data, there is potential for confounding, bias, and missing data. However, the high degree of concordance of CPRD data with the national cancer registration means misclassification of HL is likely to be low in this study, with good capture of cases. CPRD data do not include staging information, which precluded us exploring possible variation in effect estimates by stage at diagnosis. A degree of misclassification and underreporting of allergic diagnoses is likely, but this nondifferential misclassification would potentially bias toward the null, meaning that observed estimates of associations between allergic diseases and HL will be conservative. Some patients who will have contracted EBV will not experience any symptoms leading to consultation, and some who do will be misdiagnosed. These mechanisms would both similarly result in potential underestimation of effect estimates because the 2 comparator groups (cases/control subjects) become more similar artefactually, and therefore the findings for IM are likely conservative. Route of steroid administration was used as a proxy for steroid strength as a marker of a dose-response relationship (it was not possible to directly estimate cumulative steroid exposure or exact doses).

### Implications

We propose 3 potential explanations for the observed association between allergic disease and increased HL risk in early life identified in this study. The first is in support of the antigenic stimulation hypothesis for HL pathogenesis. Chronic overactivation of the immune response in patients with allergic disease over time results in randomly occurring mutations in rapidly dividing lymphocytes. These might be carcinogenic or cancer promoting, leading to HL development in predisposed subjects.

The second explanation is that allergic disease and HL development in TYAs share a common immune pathway in their development, and when regulation of this pathway is disrupted, the risk of subsequently having both conditions increases. Some studies have proposed the programmed cell death protein 1 receptor pathway and its ligands (programmed death ligands 1 and 2) as a potential culprit because its components have been linked to both allergic diseases and HL pathogenesis.^6^^,^^54^, ^55^, ^56^, ^57^, ^58^ Further studies are required to ascertain the presence and components of common underlying pathways, which, if identified, could present new targets for therapeutic intervention for these conditions.^59^

The third explanation is that therapeutic treatment for allergic diseases, such as steroids, which could themselves affect immunity, might increase a subject’s risk of having HL either directly or by increasing the risk of contracting pro-oncogenic infections, such as EBV. Disruption of the skin barrier in patients with eczema might also act in this way by increasing access to other viral pathogens. However, we observed that allergic disease was associated with increased odds of HL, even after adjusting for steroids and IM history. Further studies should explore the potential interplay between eczema, other viral infections, and HL risk.

This study showed that steroid use for any indication is associated with increased risk of HL in this patient population. There was evidence of a possible dose-response effect by route of administration, with routes of higher systemic absorption associated with greater HL risk. Interestingly, although steroid use was more frequently observed in cases than in control subjects, the effect of allergic disease on HL risk did not differ when stratifying by steroid use. This suggests the effects of steroids are not due to them being a marker of more severe allergic disease. Additionally, the association with steroids and HL persisted after adjusting for allergic diseases and other established risk factors included in the model, indicating their effect is not fully explained by these conditions. Possible explanations include that steroids might be an independent risk factor for HL, and this is a genuine causal association; however, it is more likely that steroids are a proxy for other immune diseases, which are independent risk factors for HL and occur more commonly in allergic subjects in this patient population. Previous studies have demonstrated evidence for a link between allergic diseases and other immune conditions in support of this hypothesis.^60^ Further studies are required to examine the timing, duration, and dose-response relationships between steroid exposure and HL development and the role of other immune diseases to establish their role in HL development more clearly.

### Conclusions

This study has identified allergic diseases, specifically eczema, and steroid use for any indication as risk factors for HL in early life. This is in addition to the established risk factors of immunosuppression and IM, which also cause immune dysfunction. These findings add to the growing evidence that immune dysregulation is central to the development of HL in early life and that allergic disease in childhood might increase the risk of hematologic malignancies in the future.

**Key messages**

• Allergic disease, especially eczema, is associated with increased risk of HL.
• Corticosteroid treatment is associated with increased HL risk.
• Immune system malfunction after allergic disease or immunosuppression is central to HL development.

## Supplementary data

Tables E1-E8
