# Supplementary material for: Allergic disease, corticosteroid use, and risk of Hodgkin lymphoma: A United Kingdom nationwide case-control study
Source: J Allergy Clin Immunol. 2020 Mar;145(3):868–76. doi: 10.1016/j.jaci.2019.10.033 (PMC7057259; doi:10.1016/j.jaci.2019.10.033)
Supplement: Tables E1-E8 [file mmc1.docx]

## Supplementary Tables:

**Supplementary Table S1:** Read and ICD10 codes for Hodgkin’s lymphoma

| **Read Code** | **Term** |
| --- | --- |
| **B61..00** | Hodgkin's disease |
| **B61..11** | Hodgkin lymphoma |
| **B610.00** | Hodgkin's paragranuloma |
| **B610100** | Hodgkin's paragranuloma of lymph nodes of head, face, neck |
| **B610300** | Hodgkin's paragranuloma of intra-abdominal lymph nodes |
| **B611.00** | Hodgkin's granuloma |
| **B611100** | Hodgkin's granuloma of lymph nodes of head, face and neck |
| **B612.00** | Hodgkin's sarcoma |
| **B612400** | Hodgkin's sarcoma of lymph nodes of axilla and upper limb |
| **B613.00** | Hodgkin's disease, lymphocytic-histiocytic predominance |
| **B613000** | Hodgkin's, lymphocytic-histiocytic predominance unspec site |
| **B613100** | Hodgkin's, lymphocytic-histiocytic pred of head, face, neck |
| **B613200** | Hodgkin's, lymphocytic-histiocytic pred intrathoracic nodes |
| **B613300** | Hodgkin's, lymphocytic-histiocytic pred intra-abdominal node |
| **B613500** | Hodgkin's, lymphocytic-histiocytic pred inguinal and leg |
| **B613600** | Hodgkin's, lymphocytic-histiocytic pred intrapelvic nodes |
| **B613700** | Hodgkin's, lymphocytic-histiocytic predominance of spleen |
| **B613800** | Hodgkin's, lymphocytic-histiocytic pred of multiple sites |
| **B613z00** | Hodgkin's, lymphocytic-histiocytic predominance NOS |
| **B614.00** | Hodgkin's disease, nodular sclerosis |
| **B614000** | Hodgkin's disease, nodular sclerosis of unspecified site |
| **B614100** | Hodgkin's nodular sclerosis of head, face and neck |
| **B614200** | Hodgkin's nodular sclerosis of intrathoracic lymph nodes |
| **B614300** | Hodgkin's nodular sclerosis of intra-abdominal lymph nodes |
| **B614400** | Hodgkin's nodular sclerosis of lymph nodes of axilla and arm |
| **B614700** | Hodgkin's disease, nodular sclerosis of spleen |
| **B614800** | Hodgkin's nodular sclerosis of lymph nodes of multiple sites |
| **B614z00** | Hodgkin's disease, nodular sclerosis NOS |
| **B615.00** | Hodgkin's disease, mixed cellularity |
| **B615000** | Hodgkin's disease, mixed cellularity of unspecified site |
| **B615100** | Hodgkin's mixed cellularity of lymph nodes head, face, neck |
| **B615200** | Hodgkin's mixed cellularity of intrathoracic lymph nodes |
| **B615500** | Hodgkin's mixed cellularity of lymph nodes inguinal and leg |
| **B615z00** | Hodgkin's disease, mixed cellularity NOS |
| **B616.00** | Hodgkin's disease, lymphocytic depletion |
| **B616000** | Hodgkin's lymphocytic depletion of unspecified site |
| **B616400** | Hodgkin's lymphocytic depletion lymph nodes axilla and arm |
| **B616700** | Hodgkin's disease, lymphocytic depletion of spleen |
| **B616800** | Hodgkin's lymphocytic depletion lymph nodes multiple sites |
| **B616z00** | Hodgkin's disease, lymphocytic depletion NOS |
| **B617.00** | Nodular lymphocyte predominant Hodgkin lymphoma |
| **B618.00** | Nodular sclerosis classical Hodgkin lymphoma |
| **B619.00** | Mixed cellularity classical Hodgkin lymphoma |
| **B61B.00** | Lymphocyte-rich classical Hodgkin lymphoma |
| **B61C.00** | Other classical Hodgkin lymphoma |
| **B61z.00** | Hodgkin's disease NOS |
| **B61z.11** | Hodgkin lymphoma NOS |
| **B61z000** | Hodgkin's disease NOS, unspecified site |
| **B61z100** | Hodgkin's disease NOS of lymph nodes of head, face and neck |
| **B61z200** | Hodgkin's disease NOS of intrathoracic lymph nodes |
| **B61z300** | Hodgkin's disease NOS of intra-abdominal lymph nodes |
| **B61z400** | Hodgkin's disease NOS of lymph nodes of axilla and arm |
| **B61z500** | Hodgkin's disease NOS of lymph nodes inguinal region and leg |
| **B61z700** | Hodgkin's disease NOS of spleen |
| **B61z800** | Hodgkin's disease NOS of lymph nodes of multiple sites |
| **B61zz00** | Hodgkin's disease NOS |
| **BBj..00** | [M]Hodgkin's disease |
| **BBj0.00** | [M]Hodgkin's disease NOS |
| **BBj1.00** | [M]Hodgkin's disease, lymphocytic predominance |
| **BBj1000** | [M]Hodgkin,s disease, lymphocytic predominance, diffuse |
| **BBj1100** | [M]Hodgkin,s disease, lymphocytic predominance, nodular |
| **BBj2.00** | [M]Hodgkin's disease, mixed cellularity |
| **BBj4.00** | [M]Hodgkin's disease,lymphocytic depletion,diffuse fibrosis |
| **BBj6.00** | [M]Hodgkin's disease, nodular sclerosis NOS |
| **BBj6000** | [M]Hodgkin,s disease, nodular sclerosis, lymphocytic predom |
| **BBj6100** | [M]Hodgkin,s disease, nodular sclerosis, mixed cellularity |
| **BBj6200** | [M]Hodgkin,s disease, nodular sclerosis, lymphocytic deplet |
| **BBj7.00** | [M]Hodgkin's disease, nodular sclerosis, cellular phase |
| **BBj9.00** | [M]Hodgkin's granuloma |
| **BBjz.00** | [M]Hodgkin's disease NOS |
| **ByuD000** | [X]Other Hodgkin's disease |
| **ZV10711** | [V]Personal history of Hodgkin's disease |
| **ICD10 Code** | **Term** |
| **C81** | Hodgkin lymphoma |
| **C81.0** | Nodular lymphocyte predominant Hodgkin lymphoma |
| **C81.1** | Nodular sclerosis (classical) Hodgkin lymphoma |
| **C81.2** | Mixed cellularity (classical) Hodgkin lymphoma |
| **C81.3** | Lymphocyte depleted (classical) Hodgkin lymphoma |
| **C81.4** | Lymphocyte-rich (classical) Hodgkin lymphoma |
| **C81.7** | Other (classical) Hodgkin lymphoma |
| **C81.9** | Hodgkin lymphoma unspecified |

**Supplementary Table S2:** Read and ICD10 codes for allergic disease

| **Read Code** | **Term** |
| --- | --- |
| **H33..00** | Asthma |
| **663..11** | Asthma monitoring |
| **H333.00** | Acute exacerbation of asthma |
| **H33z100** | Asthma attack |
| **H33z011** | Severe asthma attack |
| **14B4.00** | H/O: asthma |
| **H330.12** | Childhood asthma |
| **H33..11** | Bronchial asthma |
| **H330.11** | Allergic asthma |
| **663V100** | Mild asthma |
| **663V300** | Severe asthma |
| **663V000** | Occasional asthma |
| **H331.11** | Late onset asthma |
| **H33z.00** | Asthma unspecified |
| **H33zz11** | Exercise induced asthma |
| **H33z000** | Status asthmaticus NOS |
| **H331.00** | Intrinsic asthma |
| **9N1d.00** | Seen in asthma clinic |
| **H330011** | Hay fever with asthma |
| **173A.00** | Exercise induced asthma |
| **H330111** | Extrinsic asthma with asthma attack |
| **8H2P.00** | Emergency admission asthma |
| **H330.00** | Extrinsic (atopic) asthma |
| **663P.00** | Asthma limiting activities |
| **663W.00** | Asthma prophylactic medication used |
| **663U.00** | Asthma management plan given |
| **663N.00** | Asthma disturbing sleep |
| **H330.14** | Pollen asthma |
| **H33z111** | Asthma attack NOS |
| **9OJA.11** | Asthma monitored |
| **663y.00** | Number of asthma exacerbations in past year |
| **66Y5.00** | Change in asthma management plan |
| **66Y9.00** | Step up change in asthma management plan |
| **66YJ.00** | Asthma annual review |
| **8B3j.00** | Asthma medication review |
| **1J70.00** | Suspected asthma |
| **663j.00** | Asthma - currently active |
| **2126200** | Asthma resolved |
| **178..00** | Asthma trigger |
| **1O2..00** | Asthma confirmed |
| **212G.00** | Asthma resolved |
| **H33z200** | Late-onset asthma |
| **663V.00** | Asthma severity |
| **663V200** | Moderate asthma |
| **663h.00** | Asthma - currently dormant |
| **663O.00** | Asthma not disturbing sleep |
| **663Q.00** | Asthma not limiting activities |
| **663N200** | Asthma disturbs sleep frequently |
| **66YK.00** | Asthma follow-up |
| **H330000** | Extrinsic asthma without status asthmaticus |
| **H330.13** | Hay fever with asthma |
| **H33zz00** | Asthma NOS |
| **8795** | Asthma control step 2 |
| **8794** | Asthma control step 1 |
| **66YE.00** | Asthma monitoring due |
| **66YA.00** | Step down change in asthma management plan |
| **8796** | Asthma control step 3 |
| **H331111** | Intrinsic asthma with asthma attack |
| **8HTT.00** | Referral to asthma clinic |
| **66YQ.00** | Asthma monitoring by nurse |
| **663p.00** | Asthma treatment compliance unsatisfactory |
| **663n.00** | Asthma treatment compliance satisfactory |
| **9OJA.00** | Asthma monitoring check done |
| **8798** | Asthma control step 5 |
| **8797** | Asthma control step 4 |
| **H33zz12** | Allergic asthma NEC |
| **173c.00** | Occupational asthma |
| **663d.00** | Emergency asthma admission since last appointment |
| **8791** | Further asthma - drug prevent. |
| **663u.00** | Asthma causes daytime symptoms 1 to 2 times per week |
| **663e.00** | Asthma restricts exercise |
| **8CR0.00** | Asthma clinical management plan |
| **H332.00** | Mixed asthma |
| **663s.00** | Asthma never causes daytime symptoms |
| **663v.00** | Asthma causes daytime symptoms most days |
| **663f.00** | Asthma never restricts exercise |
| **663e100** | Asthma severely restricts exercise |
| **663e000** | Asthma sometimes restricts exercise |
| **H330100** | Extrinsic asthma with status asthmaticus |
| **H331000** | Intrinsic asthma without status asthmaticus |
| **8793** | Asthma control step 0 |
| **9N4Q.00** | DNA - Did not attend asthma clinic |
| **66YR.00** | Asthma monitoring by doctor |
| **663N000** | Asthma causing night waking |
| **66YP.00** | Asthma night-time symptoms |
| **663t.00** | Asthma causes daytime symptoms 1 to 2 times per month |
| **663O000** | Asthma never disturbs sleep |
| **663w.00** | Asthma limits walking up hills or stairs |
| **663x.00** | Asthma limits walking on the flat |
| **663N100** | Asthma disturbs sleep weekly |
| **H35y700** | Wood asthma |
| **663r.00** | Asthma causes night symptoms 1 to 2 times per month |
| **H334.00** | Brittle asthma |
| **U60F615** | [X] Adverse reaction to theophylline - asthma |
| **1780** | Aspirin induced asthma |
| **66YC.00** | Absent from work or school due to asthma |
| **663q.00** | Asthma daytime symptoms |
| **H331z00** | Intrinsic asthma NOS |
| **H330z00** | Extrinsic asthma NOS |
| **9OJ1.00** | Attends asthma monitoring |
| **663m.00** | Asthma accident and emergency attendance since last visit |
| **H47y000** | Detergent asthma |
| **66YZ.00** | Does not have asthma management plan |
| **TJF7300** | Adverse reaction to theophylline (asthma) |
| **H331100** | Intrinsic asthma with status asthmaticus |
| **173d.00** | Work aggravated asthma |
| **9NI8.00** | Asthma outreach clinic |
| **H35y600** | Sequoiosis (red-cedar asthma) |
| **8CMA000** | Patient has a written asthma personal action plan |
| **679J000** | Health education - asthma self management |
| **38DT.00** | Asthma control questionnaire |
| **9NNX.00** | Under care of asthma specialist nurse |
| **679J100** | Health education - structured asthma discussion |
| **66Yp.00** | Asthma review using Roy Colleg of Physicians three questions |
| **38DV.00** | Mini asthma quality of life questionnaire |
| **1787** | Asthma trigger - seasonal |
| **1781** | Asthma trigger - pollen |
| **66Yr.00** | Asthma causes symptoms most nights |
| **66Yq.00** | Asthma causes night time symptoms 1 to 2 times per week |
| **1789** | Asthma trigger - respiratory infection |
| **663P000** | Asthma limits activities 1 to 2 times per month |
| **178B.00** | Asthma trigger - exercise |
| **663P100** | Asthma limits activities 1 to 2 times per week |
| **1783** | Asthma trigger - warm air |
| **1786** | Asthma trigger - animals |
| **66Ys.00** | Asthma never causes night symptoms |
| **388t000** | Royal College Physician asthma assessment 3 question score |
| **1788** | Asthma trigger - cold air |
| **178A.00** | Asthma trigger - airborne dust |
| **1785** | Asthma trigger - damp |
| **1784** | Asthma trigger - emotion |
| **1782** | Asthma trigger - tobacco smoke |
| **663P200** | Asthma limits activities most days |
| **661N100** | Asthma self-management plan review |
| **661M100** | Asthma self-management plan agreed |
| **66Yu.00** | Number days absent from school due to asthma in past 6 month |
| **H170.11** | Hay fever - pollens |
| **H17..00** | Allergic rhinitis |
| **M12z100** | Eczema NOS |
| **M112.00** | Infantile eczema |
| **H172.00** | Allergic rhinitis due to unspecified allergen |
| **F4C1411** | Allergic conjunctivitis |
| **H17z.00** | Allergic rhinitis NOS |
| **M113.00** | Flexural eczema |
| **M12z200** | Infected eczema |
| **H17..11** | Perennial rhinitis |
| **M111.00** | Atopic dermatitis/eczema |
| **H170.00** | Allergic rhinitis due to pollens |
| **F4C0600** | Acute atopic conjunctivitis |
| **H171.00** | Allergic rhinitis due to other allergens |
| **14F1.00** | H/O: eczema |
| **M153500** | Perioral dermatitis |
| **M12z300** | Hand eczema |
| **H172.11** | Hay fever - unspecified allergen |
| **M102.11** | Pustular eczema |
| **A540.00** | Eczema herpeticum - Kaposi's varicelliform eruption |
| **M114.00** | Allergic (intrinsic) eczema |
| **M11z.00** | Atopic dermatitis NOS |
| **M12z400** | Erythrodermic eczema |
| **14B1.00** | H/O: hay fever |
| **M117.00** | Neurodermatitis - atopic |
| **26C4.00** | Nipple eczema |
| **M11..00** | Atopic dermatitis and related conditions |
| **F4C1400** | Other chronic allergic conjunctivitis |
| **M07y.11** | Pustular eczema |
| **H171.14** | Hay fever - other allergen |
| **F4C0611** | Acute allergic conjunctivitis |
| **M153511** | Circumoral dermatitis |
| **H17..12** | Allergic rhinosinusitis |
| **Myu2200** | [X]Exacerbation of eczema |
| **H170.12** | Pollinosis |
| **C391200** | Wiskott - Aldrich syndrome |
| **M115.00** | Besnier's prurigo |
| **Myu2.00** | [X]Dermatitis and eczema |
| **M153600** | Periocular dermatitis |
| **Hyu2100** | [X]Other allergic rhinitis |
| **Hyu2000** | [X]Other seasonal allergic rhinitis |
| **8HTu.00** | Referral to eczema clinic |
| **M116.11** | Brocq's neurodermatitis |
| **ICD10 Code** | **Term** |
| **B00.0** | Eczema Herpeticum |
| **D82.0** | Wiskott-Aldrich Syndrome |
| **H10.1** | Acute atopic conjunctivitis |
| **J30.1** | Allergic rhinitis due to pollen |
| **J30.2** | Other seasonal allergic rhinitis |
| **J30.3** | Other allergic rhinitis |
| **J30.4** | Allergic rhinitis, unspecified |
| **J45** | Asthma |
| **J45.0** | Predominantly allergic asthma |
| **J45.1** | Nonallergic asthma |
| **J45.8** | Mixed asthma |
| **J45.9** | Asthma unspecified |
| **J46** | Status asthmaticus |
| **L20** | atopic dermatitis |
| **L20.0** | Besnier prurigo |
| **L20.8** | Other atopic dermatitis |
| **L20.9** | atopic dermatitis, unspecified |
| **L30.9** | dermatitis, unspecified |

**Supplementary Table S3:** product codes for corticosteroids

| **Prodcode** | **Product Name** |
| --- | --- |
| **49556** | Otomize ear spray (Lexon (UK) Ltd) |
| **1018** | Otomize ear spray (Forest Laboratories UK Ltd) |
| **53529** | Generic Otomize ear spray |
| **50533** | Otomize ear spray (DE Pharmaceuticals) |
| **3507** | Modrasone 0.05%w/w Cream (Dominion Pharma) |
| **4562** | Modrasone 0.05%w/w Ointment (Dominion Pharma) |
| **8567** | Alclometasone 0.05% ointment |
| **6194** | Modrasone 0.05% cream (Teva UK Ltd) |
| **9877** | Alclometasone 0.05% cream |
| **6499** | Modrasone 0.05% ointment (Teva UK Ltd) |
| **53645** | Generic Alphosyl HC cream |
| **6140** | Alphosyl HC cream (GlaxoSmithKline Consumer Healthcare) |
| **55411** | Fluticasone propionate 50micrograms/dose / Azelastine 137micrograms/dose nasal spray |
| **55435** | Dymista 137micrograms/dose / 50micrograms/dose nasal spray (Meda Pharmaceuticals Ltd) |
| **55922** | Neomycin sulphate with nystatin bacitracin and hydrocortisone Ointment |
| **17768** | Hydroderm Ointment (MSD Thomas Morson Pharmaceuticals) |
| **12459** | Tricicatrin Ointment (Wellcome Medical Division) |
| **22880** | Beclometasone with Clioquinol cream |
| **27430** | Beclometasone with Clioquinol ointment |
| **3031** | Beclometasone 0.025% ointment |
| **3312** | Propaderm 0.025% cream (GlaxoSmithKline UK Ltd) |
| **1341** | Propaderm 0.025% ointment (GlaxoSmithKline UK Ltd) |
| **3125** | Beclometasone 0.025% cream |
| **15308** | Vivabec 50micrograms/dose nasal spray (Lexon (UK) Ltd) |
| **88** | Beclometasone 50micrograms/dose nasal spray |
| **11456** | Beclo-Aqua 50micrograms/dose nasal spray (Galen Ltd) |
| **20906** | Pollenase Hayfever 50micrograms/dose nasal spray (Peach Ethical Ltd) |
| **32873** | Beclometasone 50micrograms/dose nasal spray (Actavis UK Ltd) |
| **25999** | Beclometasone 50micrograms/dose nasal spray (A A H Pharmaceuticals Ltd) |
| **2263** | Beconase Hayfever 50micrograms/dose nasal spray (Omega Pharma Ltd) |
| **20356** | Beconase Hayfever Relief for Adults 50micrograms/dose nasal spray (Omega Pharma Ltd) |
| **888** | Beconase Aqueous 50micrograms/dose nasal spray (GlaxoSmithKline UK Ltd) |
| **19990** | Rino Clenil 50micrograms/dose nasal spray (Chiesi Ltd) |
| **55227** | Beclometasone 50micrograms/dose nasal spray (Vantage) |
| **897** | Nasobec Aqueous 50micrograms/dose nasal spray (Teva UK Ltd) |
| **18608** | Care Hayfever Relief 50micrograms/dose nasal spray (Thornton & Ross Ltd) |
| **37854** | Nasobec Hayfever 50micrograms/dose nasal spray (Teva UK Ltd) |
| **48492** | Beclometasone Hayfever Relief 50micrograms/dose nasal spray (Numark Management Ltd) |
| **53830** | Beclometasone 50micrograms/dose nasal spray (Alliance Healthcare (Distribution) Ltd) |
| **27781** | Beclometasone 50micrograms/dose nasal spray (Generics (UK) Ltd) |
| **51799** | Beconase Aqueous 50micrograms/dose nasal spray (DE Pharmaceuticals) |
| **40156** | Beclometasone hayfever 50microgram/actuation Nasal Spray (Dominion Pharma) |
| **844** | Beconase 50microgram/actuation Nasal spray suspension (Allen & Hanburys Ltd) |
| **1978** | Beclometasone 50 micrograms/actuation nasal spray |
| **17772** | Nasobec hayfever 50microgram/actuation Nasal spray suspension (Norton Consumer Distributed by Manx) |
| **20265** | Beclometasone 50microgram/actuation Nasal Spray (Numark Management Ltd) |
| **36425** | Hayfever relief 50microgram/actuation Spray (A A H Pharmaceuticals Ltd) |
| **16295** | Beclomist 50microgram/actuation Spray (Co-Pharma Ltd) |
| **8779** | Beconase 50microgram/actuation Nasal spray suspension (Allen & Hanburys Ltd) |
| **18618** | Zonivent 50microgram/actuation Spray (Ashbourne Pharmaceuticals Ltd) |
| **14294** | Qvar 50micrograms/dose Easi-Breathe inhaler (Teva UK Ltd) |
| **33258** | Beclometasone 250micrograms/dose inhaler (A A H Pharmaceuticals Ltd) |
| **4759** | Beclometasone 100microgram inhalation powder capsules |
| **2148** | Beclometasone 400microgram disc |
| **54399** | Qvar 100 Autohaler (Sigma Pharmaceuticals Plc) |
| **57589** | Becloforte 250micrograms/dose inhaler (Dowelhurst Ltd) |
| **31774** | Beclometasone 50micrograms/dose inhaler (Generics (UK) Ltd) |
| **16148** | Clenil Modulite 250micrograms/dose inhaler (Chiesi Ltd) |
| **1551** | Beclazone 250 inhaler (Teva UK Ltd) |
| **1406** | Becotide 50 inhaler (GlaxoSmithKline UK Ltd) |
| **24898** | Spacehaler BDP 100microgram/actuation Spacehaler (Celltech Pharma Europe Ltd) |
| **1242** | Beclometasone 250micrograms/dose inhaler |
| **62341** | Becotide 50 inhaler (Dowelhurst Ltd) |
| **1258** | Becotide 200 inhaler (GlaxoSmithKline UK Ltd) |
| **16158** | Clenil Modulite 50micrograms/dose inhaler (Chiesi Ltd) |
| **99** | Becotide 100 inhaler (GlaxoSmithKline UK Ltd) |
| **13037** | Pulvinal Beclometasone Dipropionate 200micrograms/dose dry powder inhaler (Chiesi Ltd) |
| **54207** | Qvar 50 inhaler (DE Pharmaceuticals) |
| **21005** | Beclometasone 250micrograms/dose inhaler CFC free |
| **2160** | Beclometasone 50micrograms/dose breath actuated inhaler |
| **46157** | Beclometasone 200 Cyclocaps (Teva UK Ltd) |
| **14321** | Beclometasone 200micrograms/dose inhaler CFC free |
| **9477** | Asmabec 100microgram/actuation Spacehaler (Celltech Pharma Europe Ltd) |
| **1951** | Becodisks 400microgram Disc (Allen & Hanburys Ltd) |
| **51681** | Qvar 100 inhaler (Sigma Pharmaceuticals Plc) |
| **47943** | Beclazone easi-breathe (roi) 100microgram/actuation Pressurised inhalation (Ivax Pharmaceuticals Ireland) |
| **25204** | Beclometasone 100micrograms/dose inhaler (A A H Pharmaceuticals Ltd) |
| **4499** | Aerobec 250microgram/actuation Pressurised inhalation (Meda Pharmaceuticals Ltd) |
| **11732** | Beclometasone 50micrograms/dose breath actuated inhaler CFC free |
| **14567** | Asmabec 250 Clickhaler (Focus Pharmaceuticals Ltd) |
| **9577** | Asmabec 50 Clickhaler (Focus Pharmaceuticals Ltd) |
| **51415** | Qvar 50 inhaler (Mawdsley-Brooks & Company Ltd) |
| **33849** | Beclometasone 100microgram/actuation Inhalation powder (Neo Laboratories Ltd) |
| **1734** | Beclometasone 100micrograms/dose breath actuated inhaler |
| **3993** | Filair Forte 250micrograms/dose inhaler (Meda Pharmaceuticals Ltd) |
| **13815** | Beclazone 100microgram/actuation Inhalation powder (Actavis UK Ltd) |
| **17654** | Easyhaler Beclometasone 200micrograms/dose dry powder inhaler (Orion Pharma (UK) Ltd) |
| **11198** | Beclometasons 50 micrograms/actuation vortex inhaler |
| **15326** | Beclometasone 100micrograms/dose inhaler CFC free |
| **1269** | Becotide 50microgram/ml Nebuliser liquid (Allen & Hanburys Ltd) |
| **5522** | Beclometasone 100micrograms/dose dry powder inhaler |
| **3927** | Filair 100 inhaler (Meda Pharmaceuticals Ltd) |
| **56493** | Qvar 50micrograms/dose Easi-Breathe inhaler (Sigma Pharmaceuticals Plc) |
| **1727** | Becotide easi-breathe 50microgram/actuation Pressurised inhalation (Allen & Hanburys Ltd) |
| **5521** | Beclometasone 200micrograms/dose dry powder inhaler |
| **3363** | Becloforte 400microgram disks with Diskhaler (GlaxoSmithKline UK Ltd) |
| **35580** | Beclometasone 100microgram inhalation powder blisters with device |
| **1885** | Beclazone 200 inhaler (Teva UK Ltd) |
| **16584** | Beclometasone 50micrograms/dose inhaler CFC free |
| **9233** | Beclometasone 200microgram inhalation powder capsules |
| **14757** | Pulvinal Beclometasone Dipropionate 100micrograms/dose dry powder inhaler (Chiesi Ltd) |
| **3947** | Becotide 100microgram Rotacaps (GlaxoSmithKline UK Ltd) |
| **35288** | Beclometasone 400microgram inhalation powder blisters |
| **35293** | Beclometasone 200microgram inhalation powder blisters with device |
| **28761** | Spacehaler BDP 50microgram/actuation Spacehaler (Celltech Pharma Europe Ltd) |
| **30238** | Beclometasone 50microgram/actuation Pressurised inhalation (Approved Prescription Services Ltd) |
| **1725** | Beclazone 50 Easi-Breathe inhaler (Teva UK Ltd) |
| **53480** | Qvar 100 Autohaler (Stephar (U.K.) Ltd) |
| **18848** | Qvar 100micrograms/dose Easi-Breathe inhaler (Teva UK Ltd) |
| **2992** | Beclazone 50 inhaler (Teva UK Ltd) |
| **19389** | Asmabec 50microgram/actuation Spacehaler (Celltech Pharma Europe Ltd) |
| **13290** | Clenil Modulite 100micrograms/dose inhaler (Chiesi Ltd) |
| **34919** | Beclometasone 50micrograms/dose inhaler (A A H Pharmaceuticals Ltd) |
| **1100** | Beclazone 100 inhaler (Teva UK Ltd) |
| **28073** | Beclometasone 250microgram/actuation Pressurised inhalation (Approved Prescription Services Ltd) |
| **9921** | Beclometasone 100micrograms/dose breath actuated inhaler CFC free |
| **2600** | Beclometasone 250micrograms/dose breath actuated inhaler |
| **1243** | Beclazone 250 Easi-Breathe inhaler (Teva UK Ltd) |
| **35430** | Becodisks 200microgram with Diskhaler (GlaxoSmithKline UK Ltd) |
| **41412** | Beclometasone 400micrograms/actuation inhaler |
| **41269** | Beclometasone 400 Cyclocaps (Teva UK Ltd) |
| **2892** | Becloforte 400microgram disks (GlaxoSmithKline UK Ltd) |
| **35118** | Becodisks 400microgram with Diskhaler (GlaxoSmithKline UK Ltd) |
| **7964** | Beclometasone 50micrograms/ml nebuliser suspension |
| **34428** | Beclometasone 50microgram/actuation Inhalation powder (Neo Laboratories Ltd) |
| **9571** | Beclometasone 250micrograms/actuation vortex inhaler |
| **1552** | Becloforte easi-breathe 250microgram/actuation Pressurised inhalation (Allen & Hanburys Ltd) |
| **19031** | Bdp 100microgram/actuation Spacehaler (Celltech Pharma Europe Ltd) |
| **34794** | Beclometasone 200micrograms/dose inhaler (A A H Pharmaceuticals Ltd) |
| **20825** | Spacehaler BDP 250microgram/actuation Spacehaler (Celltech Pharma Europe Ltd) |
| **2229** | Becodisks 100microgram Disc (Allen & Hanburys Ltd) |
| **896** | Becotide easi-breathe 100microgram/actuation Pressurised inhalation (Allen & Hanburys Ltd) |
| **10090** | Beclometasone 50micrograms/actuation extrafine particle cfc free inhaler |
| **5804** | Beclometasone 250micrograms/dose dry powder inhaler |
| **56471** | Becodisks 200microgram (Mawdsley-Brooks & Company Ltd) |
| **61664** | Clenil Modulite 250micrograms/dose inhaler (Waymade Healthcare Plc) |
| **34739** | Beclometasone 50micrograms/dose inhaler (Teva UK Ltd) |
| **48340** | Clenil Modulite 100micrograms/dose inhaler (Mawdsley-Brooks & Company Ltd) |
| **35408** | Becodisks 100microgram (GlaxoSmithKline UK Ltd) |
| **3743** | Filair 50 inhaler (Meda Pharmaceuticals Ltd) |
| **28640** | Beclometasone 100microgram/actuation Inhalation powder (Actavis UK Ltd) |
| **38** | Beclometasone 100micrograms/dose inhaler |
| **51480** | Qvar 100 Autohaler (DE Pharmaceuticals) |
| **11497** | Beclometasone 400micrograms/dose dry powder inhaler |
| **7653** | Beclometasone 400microgram inhalation powder capsules |
| **29325** | Beclometasone 250micrograms/dose inhaler (Generics (UK) Ltd) |
| **21482** | Beclometasone 100micrograms/dose inhaler (Generics (UK) Ltd) |
| **35652** | Beclometasone 100microgram inhalation powder blisters |
| **26063** | Beclometasone 100micrograms/dose inhaler (Teva UK Ltd) |
| **35107** | Beclometasone 400microgram inhalation powder blisters with device |
| **35071** | Becodisks 200microgram (GlaxoSmithKline UK Ltd) |
| **4365** | Beclometasone 100micrograms disc |
| **39200** | AeroBec Forte 250 Autohaler (Meda Pharmaceuticals Ltd) |
| **18394** | Bdp 50microgram/actuation Spacehaler (Celltech Pharma Europe Ltd) |
| **895** | Beclazone 100 Easi-Breathe inhaler (Teva UK Ltd) |
| **51234** | Qvar 100 inhaler (Waymade Healthcare Plc) |
| **5992** | Beclometasone 50micrograms/dose dry powder inhaler |
| **3119** | Becloforte integra 250microgram/actuation Inhaler with compact spacer (Glaxo Laboratories Ltd) |
| **14524** | Bdp 250microgram/actuation Spacehaler (Celltech Pharma Europe Ltd) |
| **50287** | Qvar 100 inhaler (DE Pharmaceuticals) |
| **3075** | Becotide 400microgram Rotacaps (GlaxoSmithKline UK Ltd) |
| **3018** | Beclometasone 50micrograms/dose inhaler |
| **3150** | Beclometasone 100micrograms/actuation extrafine particle cfc free inhaler |
| **19401** | Beclometasone 250micrograms/actuation inhaler and compact spacer |
| **49367** | Clenil Modulite 50micrograms/dose inhaler (Mawdsley-Brooks & Company Ltd) |
| **4413** | Qvar 100 Autohaler (Teva UK Ltd) |
| **32874** | Beclometasone 50microgram/actuation Inhalation powder (Actavis UK Ltd) |
| **30210** | Beclometasone 250micrograms/dose inhaler (Teva UK Ltd) |
| **2335** | Qvar 100 inhaler (Teva UK Ltd) |
| **4601** | Asmabec 100 Clickhaler (Focus Pharmaceuticals Ltd) |
| **16151** | Clenil Modulite 200micrograms/dose inhaler (Chiesi Ltd) |
| **2159** | AeroBec 50 Autohaler (Meda Pharmaceuticals Ltd) |
| **52806** | Qvar 100 Autohaler (Lexon (UK) Ltd) |
| **35106** | Becodisks 100microgram with Diskhaler (GlaxoSmithKline UK Ltd) |
| **1537** | Becotide 200microgram Rotacaps (GlaxoSmithKline UK Ltd) |
| **1259** | Beclometasone 200micrograms/dose inhaler |
| **9599** | Beclazone 50microgram/actuation Inhalation powder (Actavis UK Ltd) |
| **1236** | Becloforte 250micrograms/dose inhaler (GlaxoSmithKline UK Ltd) |
| **3546** | Qvar 50 inhaler (Teva UK Ltd) |
| **14736** | Pulvinal Beclometasone Dipropionate 400micrograms/dose dry powder inhaler (Chiesi Ltd) |
| **35113** | Beclometasone 200microgram inhalation powder blisters |
| **50129** | Qvar 100micrograms/dose Easi-Breathe inhaler (DE Pharmaceuticals) |
| **8111** | Becloforte vm 250microgram/actuation VM pack (Allen & Hanburys Ltd) |
| **3220** | Qvar 50 Autohaler (Teva UK Ltd) |
| **883** | Becodisks 200microgram Disc (Allen & Hanburys Ltd) |
| **35299** | Becodisks 400microgram (GlaxoSmithKline UK Ltd) |
| **2893** | Beclometasone 200micrograms disc |
| **4803** | Beclazone 250microgram/actuation Inhalation powder (Actavis UK Ltd) |
| **56462** | Becodisks 400microgram (Waymade Healthcare Plc) |
| **34315** | Beclometasone 250microgram/actuation Inhalation powder (Actavis UK Ltd) |
| **1861** | AeroBec 100 Autohaler (Meda Pharmaceuticals Ltd) |
| **34859** | Beclometasone 250microgram/actuation Inhalation powder (Neo Laboratories Ltd) |
| **27679** | Beclometasone 100microgram/actuation Pressurised inhalation (Approved Prescription Services Ltd) |
| **15706** | Beclometasone 100 micrograms/actuation vortex inhaler |
| **14590** | Asmabec 250microgram/actuation Spacehaler (Celltech Pharma Europe Ltd) |
| **48709** | Qvar 100micrograms/dose Easi-Breathe inhaler (Sigma Pharmaceuticals Plc) |
| **19376** | Beclometasone 200micrograms with Salbutamol 400micrograms inhalation capsules |
| **3556** | Beclometasone 50micrograms with salbutamol 100micrograms/inhalation inhaler |
| **19121** | Beclometasone 100micrograms with Salbutamol 200micrograms inhalation capsules |
| **37203** | Beclometasone 5mg gastro-resistant modified-release tablets |
| **39067** | Clipper 5mg gastro-resistant modified-release tablets (Chiesi Ltd) |
| **37432** | Fostair 100micrograms/dose / 6micrograms/dose inhaler (Chiesi Ltd) |
| **37470** | Beclometasone 100micrograms/dose / Formoterol 6micrograms/dose inhaler CFC free |
| **16625** | Ventide Rotacaps (GlaxoSmithKline UK Ltd) |
| **14561** | Salbutamol 400microgram / Beclometasone 200microgram inhalation powder capsules |
| **18456** | Salbutamol 200microgram / Beclometasone 100microgram inhalation powder capsules |
| **18484** | Ventide Paediatric Rotacaps (GlaxoSmithKline UK Ltd) |
| **8787** | Hydrocortisone with nystatin dimeticone and benzalkonium chloride Ointment |
| **7015** | Hydrocortisone with nystatin dimeticone and benzalkonium chloride Cream |
| **3223** | Quinoderm with hydrocortisone 1% Cream (Quinoderm Ltd) |
| **9026** | Lederspan 20mg/ml Injection (Wyeth Pharmaceuticals) |
| **16815** | Triamcinolone hexacetonide 20mg/ml Injection |
| **61413** | Anusol Soothing Relief suppositories (McNeil Products Ltd) |
| **1992** | Anusol HC suppositories (McNeil Products Ltd) |
| **2581** | Anusol Plus HC suppositories (McNeil Products Ltd) |
| **57716** | Generic Anusol HC suppositories |
| **59369** | Generic Anugesic-HC cream |
| **2608** | Anugesic-HC cream (Pfizer Ltd) |
| **57157** | Generic Anusol HC ointment |
| **1796** | Anusol HC ointment (McNeil Products Ltd) |
| **6186** | Anusol Plus HC ointment (McNeil Products Ltd) |
| **62268** | Anusol Soothing Relief ointment (McNeil Products Ltd) |
| **11149** | Betnelan 500microgram tablets (Focus Pharmaceuticals Ltd) |
| **10864** | Betamethasone 500microgram tablets |
| **28295** | Diprosone duopack Cream (Manufacturer unknown) |
| **21469** | Diprosone duopack Ointment (Manufacturer unknown) |
| **7753** | Betamethasone dipropionate 0.05% cream |
| **3727** | Diprosone 0.05% cream (Merck Sharp & Dohme Ltd) |
| **3761** | Diprosone 0.05% lotion (Merck Sharp & Dohme Ltd) |
| **7324** | Betamethasone dipropionate 0.05% scalp lotion |
| **7820** | Betamethasone dipropionate 0.05% ointment |
| **1869** | Diprosone 0.05% ointment (Merck Sharp & Dohme Ltd) |
| **62138** | Dovobet gel (Waymade Healthcare Plc) |
| **38959** | Calcipotriol 0.005% / Betamethasone dipropionate 0.05% gel |
| **43946** | Dovobet gel (LEO Pharma) |
| **38957** | Xamiol gel (LEO Pharma) |
| **23035** | Betamethasone dipropionate with fluocinonide, gentamicin with salicylic acid ointment |
| **32022** | Vipsogal Ointment (Harley Street Supplies Ltd) |
| **3192** | Betamethasone dipropionate 0.05% / Salicylic acid 3% ointment |
| **52027** | Diprosalic 0.05%/3% ointment (DE Pharmaceuticals) |
| **1325** | Diprosalic 0.05%/3% ointment (Merck Sharp & Dohme Ltd) |
| **49852** | Diprosalic 0.05%/3% ointment (Sigma Pharmaceuticals Plc) |
| **7396** | Betnesol 0.10% Ear/eye/nose drops solution (UCB Pharma Ltd) |
| **5554** | Betamethasone sodium phosphate 0.1% nasal drops |
| **2981** | Betamethasone sodium phosphate 0.1% eye drops |
| **885** | Betnesol 0.10% Eye drops (UCB Pharma Ltd) |
| **4664** | Betnesol 0.10% Nasal drops (UCB Pharma Ltd) |
| **6960** | Vista-methasone Nasal drops (Martindale Pharmaceuticals Ltd) |
| **7180** | Vista-methasone Ear drops (Martindale Pharmaceuticals Ltd) |
| **14973** | Vistamethasone 0.1% ear/eye/nose drops (Martindale Pharmaceuticals Ltd) |
| **42937** | Betnesol 0.1% eye/ear/nose drops (Focus Pharmaceuticals Ltd) |
| **7363** | Betamethasone 0.1% ear/eye/nose drops |
| **1744** | Vista-methasone Eye drops (Martindale Pharmaceuticals Ltd) |
| **5332** | Betnesol 0.10% Ear drops (UCB Pharma Ltd) |
| **5125** | Betamethasone sodium phosphate 0.1% ear drops |
| **8529** | Betamethasone 0.1% eye ointment |
| **2011** | Betnesol 0.1% eye ointment (Focus Pharmaceuticals Ltd) |
| **22577** | Betnesol 4mg/1ml solution for injection ampoules (Focus Pharmaceuticals Ltd) |
| **15717** | Betamethasone 4mg/1ml solution for injection ampoules |
| **1971** | Betnesol 500microgram soluble tablets (Focus Pharmaceuticals Ltd) |
| **50225** | Betnesol 500microgram soluble tablets (Waymade Healthcare Plc) |
| **7286** | Betamethasone 500microgram soluble tablets sugar free |
| **18898** | Vistamethasone N ear/eye/nose drops (Martindale Pharmaceuticals Ltd) |
| **13269** | Betamethasone 0.1% / Neomycin 0.5% ear/eye/nose drops |
| **7321** | Betnesol -n Ear/eye/nose drops solution (UCB Pharma Ltd) |
| **43066** | Betnesol-N ear/eye/nose drops (Focus Pharmaceuticals Ltd) |
| **40393** | Coal tar solution in Betnovate RD (HRI) ointment 5% |
| **40387** | Coal tar 5% Ointment (Royal Free Hospital) |
| **54461** | Coal tar solution in Betnovate RD (Royal Free Hosp) 5% ointment |
| **53165** | Coal tar solution with Betamethasone 0.1% ointment in white soft paraffin (BCM) 3% with 50% |
| **28424** | Betamethasone 0.10% Cream (Futuna Ltd) |
| **29329** | Betamethasone 0.10% Ointment (Futuna Ltd) |
| **36867** | Betamethasone valerate 0.1% ointment in white soft paraffin ointment 10% |
| **3366** | Betamethasone valerate 0.025% cream |
| **49208** | Betnovate 0.1% ointment (Mawdsley-Brooks & Company Ltd) |
| **2573** | Betacap 0.1% scalp application (Dermal Laboratories Ltd) |
| **48615** | Betnovate 0.1% scalp application (Mawdsley-Brooks & Company Ltd) |
| **53408** | Betnovate 0.1% scalp application (Lexon (UK) Ltd) |
| **985** | Betamethasone valerate 0.1% scalp application |
| **905** | Betnovate 0.1% cream (GlaxoSmithKline UK Ltd) |
| **1914** | Betamethasone valerate 0.1% ointment |
| **6801** | Betamethasone valerate 0.025% ointment |
| **1022** | Betnovate 0.1% ointment (GlaxoSmithKline UK Ltd) |
| **42085** | Betamethasone valerate 0.1% ointment (A A H Pharmaceuticals Ltd) |
| **40973** | Betamethasone valerate 2.25mg medicated plasters |
| **55487** | Betamethasone valerate 0.1% ointment (Teva UK Ltd) |
| **1206** | Betamethasone valerate 0.1% cream |
| **60836** | Betamethasone valerate 0.025% ointment (A A H Pharmaceuticals Ltd) |
| **34279** | Betamethasone valerate 0.1% cream (A A H Pharmaceuticals Ltd) |
| **3088** | Betamethasone valerate 0.1% lotion |
| **57787** | Audavate RD 0.025% ointment (Auden McKenzie (Pharma Division) Ltd) |
| **61772** | Betamethasone valerate 0.025% cream (A A H Pharmaceuticals Ltd) |
| **984** | Betnovate 0.1% scalp application (GlaxoSmithKline UK Ltd) |
| **50488** | Betnovate 0.1% scalp application (Sigma Pharmaceuticals Plc) |
| **49936** | Betnovate 0.1% scalp application (DE Pharmaceuticals) |
| **1433** | Betnovate RD 0.025% ointment (GlaxoSmithKline UK Ltd) |
| **58388** | Audavate 0.1% ointment (Auden McKenzie (Pharma Division) Ltd) |
| **2742** | Bettamousse 0.1% cutaneous foam (Focus Pharmaceuticals Ltd) |
| **57519** | Betnovate 0.1% ointment (Dowelhurst Ltd) |
| **40858** | Betesil 2.25mg medicated plasters (Genus Pharmaceuticals Ltd) |
| **1472** | Betnovate RD 0.025% cream (GlaxoSmithKline UK Ltd) |
| **49522** | Betnovate 0.1% scalp application (Waymade Healthcare Plc) |
| **60189** | Audavate RD 0.025% cream (Auden McKenzie (Pharma Division) Ltd) |
| **1536** | Betnovate 0.1% lotion (GlaxoSmithKline UK Ltd) |
| **49478** | Betnovate 0.1% cream (Mawdsley-Brooks & Company Ltd) |
| **6799** | Betamethasone 0.1% foam |
| **41386** | Betamethasone 0.1% ointment in emollient cream 50:50 |
| **7724** | Betamethasone valerate 100micrograms/actuation inhaler |
| **3065** | Bextasol Inhalation powder (Allen & Hanburys Ltd) |
| **9109** | Betnovate Suppository (Glaxo Laboratories Ltd) |
| **1737** | Betamethasone valerate 0.1% / Fusidic acid 2% cream |
| **37921** | Fucibet Lipid cream (LEO Pharma) |
| **642** | Fucibet cream (LEO Pharma) |
| **3148** | Betnovate rectal ointment (GlaxoSmithKline UK Ltd) |
| **1989** | Betamethasone valerate 0.1% / Neomycin 0.5% cream |
| **1533** | Betnovate-N cream (Chemidex Pharma Ltd) |
| **62067** | Salicylic acid 5% in Betamethasone valerate 0.1% ointment |
| **17100** | Budesonide 0.025% cream |
| **29439** | Budesonide 0.025% ointment |
| **16654** | Preferid 0.025% Ointment (Yamanouchi Pharma Ltd) |
| **8927** | Preferid 0.025% Cream (Yamanouchi Pharma Ltd) |
| **6496** | Budesonide 100micrograms/dose nasal spray |
| **6546** | Rhinocort Aqua 64 nasal spray (AstraZeneca UK Ltd) |
| **34875** | Budesonide 100micrograms/dose nasal spray (Generics (UK) Ltd) |
| **25035** | Budesonide 50micrograms.actuation nasal spray |
| **2037** | Rhinocort 50microgram/actuation Nasal spray (AstraZeneca UK Ltd) |
| **2049** | Rhinocort Aqua 100micrograms/dose nasal spray (AstraZeneca UK Ltd) |
| **56450** | Budesonide 100micrograms/dose nasal spray (Sigma Pharmaceuticals Plc) |
| **6978** | Budesonide 64micrograms/dose nasal spray |
| **1959** | Pulmicort 0.5mg Respules (AstraZeneca UK Ltd) |
| **61975** | Budesonide 500micrograms/2ml nebuliser liquid unit dose vials (Almus Pharmaceuticals Ltd) |
| **49711** | Pulmicort 200micrograms/dose inhaler (AstraZeneca UK Ltd) |
| **454** | Pulmicort 200microgram Inhaler (AstraZeneca UK Ltd) |
| **17670** | Easyhaler Budesonide 100micrograms/dose dry powder inhaler (Orion Pharma (UK) Ltd) |
| **56498** | Pulmicort 200 Turbohaler (Waymade Healthcare Plc) |
| **1642** | Budesonide 400micrograms/dose dry powder inhaler |
| **4801** | Budesonide 500micrograms/2ml nebuliser liquid unit dose vials |
| **35510** | Budesonide 200micrograms/dose dry powder inhalation cartridge with device |
| **2092** | Budesonide 200micrograms/dose dry powder inhaler |
| **3570** | Budesonide 200micrograms/actuation refill canister |
| **16054** | Budesonide 200micrograms/actuation breath actuated powder inhaler |
| **1956** | Pulmicort 1mg Respules (AstraZeneca UK Ltd) |
| **908** | Pulmicort 400 Turbohaler (AstraZeneca UK Ltd) |
| **35724** | Budelin Novolizer 200micrograms/dose inhalation powder refill (Meda Pharmaceuticals Ltd) |
| **7788** | Budesonide 100micrograms/dose dry powder inhaler |
| **14700** | Budesonide 400micrograms/actuation inhaler |
| **8433** | Budesonide 100micrograms/actuation inhaler |
| **1680** | Pulmicort LS 50micrograms/dose inhaler (AstraZeneca UK Ltd) |
| **2125** | Pulmicort 200microgram Refill canister (AstraZeneca UK Ltd) |
| **35631** | Budelin Novolizer 200micrograms/dose inhalation powder (Meda Pharmaceuticals Ltd) |
| **960** | Pulmicort 100 Turbohaler (AstraZeneca UK Ltd) |
| **4942** | Budesonide 1mg/2ml nebuliser liquid unit dose vials |
| **23741** | Novolizer budesonide 200microgram/actuation Pressurised inhalation (Meda Pharmaceuticals Ltd) |
| **35602** | Budesonide 200micrograms/dose dry powder inhalation cartridge |
| **60937** | Pulmicort 200 Turbohaler (Dowelhurst Ltd) |
| **50037** | Pulmicort 0.5mg Respules (Waymade Healthcare Plc) |
| **40057** | Pulmicort 200micrograms/dose inhaler CFC free (AstraZeneca UK Ltd) |
| **956** | Pulmicort 200 Turbohaler (AstraZeneca UK Ltd) |
| **27188** | Easyhaler Budesonide 200micrograms/dose dry powder inhaler (Orion Pharma (UK) Ltd) |
| **947** | Budesonide 50micrograms/actuation refill canister |
| **52732** | Pulmicort 0.5mg Respules (Necessity Supplies Ltd) |
| **30649** | Easyhaler Budesonide 400micrograms/dose dry powder inhaler (Orion Pharma (UK) Ltd) |
| **4545** | Pulmicort LS 50microgram Refill canister (AstraZeneca UK Ltd) |
| **6095** | Budesonide 3mg gastro-resistant capsules |
| **60946** | Entocort CR 3mg capsules (Waymade Healthcare Plc) |
| **16525** | Budenofalk 3mg gastro-resistant capsules (Dr. Falk Pharma UK Ltd) |
| **18093** | Entocort 2mg/100ml enema (AstraZeneca UK Ltd) |
| **1380** | Entocort CR 3mg capsules (AstraZeneca UK Ltd) |
| **35595** | Budesonide 2mg foam enema |
| **11242** | Budesonide 2mg/100ml enema |
| **36630** | Budenofalk 2mg foam enema (Dr. Falk Pharma UK Ltd) |
| **51997** | Budesonide 9mg gastro-resistant granules sachets |
| **56144** | Budenofalk 9mg gastro-resistant granules sachets (Dr. Falk Pharma UK Ltd) |
| **3898** | Budesonide 3mg gastro-resistant modified-release capsules |
| **10321** | Budesonide 400microgram inhalation powder capsules |
| **39102** | Budesonide 100micrograms/dose inhaler CFC free |
| **909** | Budesonide 200micrograms/dose inhaler |
| **47225** | Budesonide 9mg gastro-resistant granules sachets |
| **39879** | Budesonide 200micrograms/dose inhaler CFC free |
| **39099** | Pulmicort 100micrograms/dose inhaler CFC free (AstraZeneca UK Ltd) |
| **18537** | Budesonide 200microgram inhalation powder capsules |
| **959** | Budesonide 50micrograms/dose inhaler |
| **48088** | Budenofalk 9mg gastro-resistant granules sachets (Dr. Falk Pharma UK Ltd) |
| **50739** | Symbicort 400/12 Turbohaler (Mawdsley-Brooks & Company Ltd) |
| **6796** | Budesonide 200micrograms/dose / Formoterol 6micrograms/dose dry powder inhaler |
| **61782** | DuoResp Spiromax 160micrograms/dose / 4.5micrograms/dose dry powder inhaler (Teva UK Ltd) |
| **51759** | Symbicort 200/6 Turbohaler (Mawdsley-Brooks & Company Ltd) |
| **7013** | Symbicort 100/6 Turbohaler (AstraZeneca UK Ltd) |
| **51570** | Symbicort 200/6 Turbohaler (DE Pharmaceuticals) |
| **53491** | Symbicort 200/6 Turbohaler (Sigma Pharmaceuticals Plc) |
| **10218** | Budesonide 100micrograms/dose / Formoterol 6micrograms/dose dry powder inhaler |
| **6325** | Symbicort 200/6 Turbohaler (AstraZeneca UK Ltd) |
| **6780** | Symbicort 400/12 Turbohaler (AstraZeneca UK Ltd) |
| **53237** | Symbicort 400/12 Turbohaler (DE Pharmaceuticals) |
| **6746** | Budesonide 400micrograms/dose / Formoterol 12micrograms/dose dry powder inhaler |
| **50945** | Symbicort 100/6 Turbohaler (Mawdsley-Brooks & Company Ltd) |
| **61666** | DuoResp Spiromax 320micrograms/dose / 9micrograms/dose dry powder inhaler (Teva UK Ltd) |
| **49114** | Symbicort 100/6 Turbohaler (Sigma Pharmaceuticals Plc) |
| **22684** | Hydrocortisone with calamine Cream |
| **3529** | Hydrocal 1% Cream (Bioglan Laboratories Ltd) |
| **8727** | Eczederm with hydrocortisone Cream (Quinoderm Ltd) |
| **6587** | Calcipotriol 0.005% / Betamethasone dipropionate 0.05% ointment |
| **5828** | Dovobet ointment (LEO Pharma) |
| **51816** | Dovobet ointment (Sigma Pharmaceuticals Plc) |
| **52603** | Dovobet ointment (DE Pharmaceuticals) |
| **4901** | Nystatin with hydrocortisone and chlorhexidine Cream |
| **13939** | Nystatin with hydrocortisone and chlorhexidine Ointment |
| **17513** | Hydrocortisone with nystatin and chlorhexidine Ointment |
| **7826** | Hydrocortisone with nystatin and chlorhexidine Cream |
| **8413** | Locoid C cream (Astellas Pharma Ltd) |
| **3933** | Locoid C ointment (Astellas Pharma Ltd) |
| **11200** | Chlorquinaldol 3% / Hydrocortisone butyrate 0.1% ointment |
| **8503** | Chlorquinaldol 3% / Hydrocortisone butyrate 0.1% cream |
| **21755** | Triamcinolone acetonide with chlortetracycline hc Cream |
| **19905** | Beclometasone with Chlortetracycline Hcl ointment |
| **1606** | Aureocort Cream (Wyeth Pharmaceuticals) |
| **1340** | Propaderm -A Ointment (Glaxo Laboratories Ltd) |
| **38074** | Chlortetracycline with hydrocortisone cream |
| **21224** | Alvesco 80 inhaler (Takeda UK Ltd) |
| **7356** | Ciclesonide 80micrograms/dose inhaler CFC free |
| **6839** | Alvesco 160 inhaler (Takeda UK Ltd) |
| **10102** | Ciclesonide 160micrograms/dose inhaler CFC free |
| **58705** | Generic Ultraproct ointment |
| **8778** | Ultraproct ointment (Meadow Laboratories Ltd) |
| **4958** | Ultraproct suppositories (Meadow Laboratories Ltd) |
| **1044** | Proctosedyl suppositories (Sanofi) |
| **9544** | Uniroid HC ointment (Chemidex Pharma Ltd) |
| **915** | Proctosedyl ointment (Sanofi) |
| **10050** | Uniroid HC suppositories (Chemidex Pharma Ltd) |
| **50597** | Proctosedyl ointment (DE Pharmaceuticals) |
| **5528** | Cinchocaine 0.5% / Hydrocortisone 0.5% ointment |
| **5529** | Cinchocaine 5mg / Hydrocortisone 5mg suppositories |
| **14695** | Prednisolone 1.3mg with cinchocaine 1mg suppositories |
| **6073** | Prednisolone 1.9mg/g with cinchocaine 5mg/g ointment |
| **2762** | Scheriproct ointment (Bayer Plc) |
| **4657** | Cinchocaine 0.5% / Prednisolone 0.19% ointment |
| **29385** | Clioquinol 3% with Fluocinolone 0.025% ointment |
| **19297** | Clioquinol 3% with Fluocinolone 0.025% cream |
| **10231** | Clioquinol 3% with betamethasone valerate 0.1% cream |
| **11597** | Clioquinol 3% with Betamethasone valerate 0.1% ointment |
| **2301** | Clioquinol 3% with Hydrocortisone 1% ointment |
| **2705** | Clioquinol 3% with Hydrocortisone 1% cream |
| **4106** | Betamethasone valerate 0.1% / Clioquinol 3% cream |
| **1583** | Betnovate-C cream (Chemidex Pharma Ltd) |
| **13211** | Betamethasone valerate 0.1% / Clioquinol 3% ointment |
| **57474** | Betamethasone valerate 0.1% / Clioquinol 3% cream (A A H Pharmaceuticals Ltd) |
| **1475** | Betnovate-C ointment (Chemidex Pharma Ltd) |
| **56871** | Betamethasone valerate 0.1% / Clioquinol 3% ointment (A A H Pharmaceuticals Ltd) |
| **57902** | Betamethasone valerate 0.1% / Clioquinol 3% ointment (Essential Generics Ltd) |
| **61725** | Flumetasone 0.02% / Clioquinol 1% ear drops (Alliance Healthcare (Distribution) Ltd) |
| **6625** | Flumetasone 0.02% / Clioquinol 1% ear drops |
| **1201** | Locorten Vioform ear drops (AMCo) |
| **18095** | Fluocinolone acetonide 0.025% / Clioquinol 3% cream |
| **8626** | Synalar C ointment (Derma UK Ltd) |
| **12742** | Fluocinolone acetonide 0.025% / Clioquinol 3% ointment |
| **7986** | Synalar C cream (Derma UK Ltd) |
| **12457** | Barquinol hc Cream (Rpr / Fisons) |
| **11505** | Clioquinol 3% / Hydrocortisone 1% cream |
| **914** | Vioform-Hydrocortisone cream (Novartis Consumer Health UK Ltd) |
| **7637** | Clobetasol propionate with neomycin ointment |
| **4940** | Clobetasol propionate 0.05% with neomycin 0.5% with nystatin 100000 units/g cream |
| **46864** | Clobetasol propionate with neomycin with nystatin 0.05% with 0.5% with 100000 unit/g Cream (Essential Generics Ltd) |
| **8231** | Clobetasol propionate with neomycin cream |
| **1955** | Dermovate -nn Ointment (Glaxo Laboratories Ltd) |
| **777** | Dermovate -nn Cream (Glaxo Laboratories Ltd) |
| **47728** | Clobetasol propionate with neomycin with nystatin 0.05% with 0.5% with 100000 unit/g ointment (Essential Generics Ltd) |
| **9244** | Clobetasol propionate with neomycin with nystatin 0.05% with 0.5% with 100000 unit/g ointment |
| **41388** | Clobetasol propionate 25% ointment with coal tar solution 5% in white soft paraffin |
| **48893** | Dermovate 0.05% cream (Waymade Healthcare Plc) |
| **49304** | Dermovate 0.05% cream (Mawdsley-Brooks & Company Ltd) |
| **49780** | Dermovate 0.05% scalp application (Sigma Pharmaceuticals Plc) |
| **311** | Dermovate 0.05% cream (GlaxoSmithKline UK Ltd) |
| **53410** | Dermovate 0.05% cream (DE Pharmaceuticals) |
| **50623** | Dermovate 0.05% cream (Sigma Pharmaceuticals Plc) |
| **51862** | Dermovate 0.05% scalp application (Lexon (UK) Ltd) |
| **50610** | Dermovate 0.05% cream (Necessity Supplies Ltd) |
| **2212** | Dermovate 0.05% scalp application (GlaxoSmithKline UK Ltd) |
| **1023** | Dermovate 0.05% ointment (GlaxoSmithKline UK Ltd) |
| **52360** | Dermovate 0.05% scalp application (Waymade Healthcare Plc) |
| **21580** | Clarelux 500micrograms/g foam (Pierre Fabre Dermo-Cosmetique) |
| **13286** | Clobetasol 500micrograms/g foam |
| **54142** | ClobaDerm 0.05% cream (Auden McKenzie (Pharma Division) Ltd) |
| **48849** | Dermovate 0.05% ointment (Waymade Healthcare Plc) |
| **5876** | Clobetasol 0.05% scalp application |
| **1682** | Clobetasol 0.05% cream |
| **36014** | Etrivex 500micrograms/g shampoo (Galderma (UK) Ltd) |
| **36226** | Clobetasol 500micrograms/g shampoo |
| **2983** | Clobetasol 0.05% ointment |
| **54818** | ClobaDerm 0.05% ointment (Auden McKenzie (Pharma Division) Ltd) |
| **61104** | Clobetasol 500microgram / Neomycin 5mg / Nystatin 100,000units/g cream (Mawdsley-Brooks & Company Ltd) |
| **53762** | Clobetasol 500microgram / Neomycin 5mg / Nystatin 100,000units/g cream (A A H Pharmaceuticals Ltd) |
| **50819** | Clobetasol 500microgram / Neomycin 5mg / Nystatin 100,000units/g cream |
| **60921** | Clobetasol 500microgram / Neomycin 5mg / Nystatin 100,000units/g ointment |
| **53839** | Clobetasol 500microgram / Neomycin 5mg / Nystatin 100,000units/g ointment (A A H Pharmaceuticals Ltd) |
| **49462** | Clobetasol 500microgram / Neomycin 5mg / Nystatin 100,000units/g ointment (Alliance Healthcare (Distribution) Ltd) |
| **48830** | Clobetasol 500microgram / Neomycin 5mg / Nystatin 100,000units/g cream (Alliance Healthcare (Distribution) Ltd) |
| **50969** | Clobetasol 500microgram / Neomycin 5mg / Nystatin 100,000units/g ointment (Essential Generics Ltd) |
| **49687** | Clobetasol 500microgram / Neomycin 5mg / Nystatin 100,000units/g cream (Essential Generics Ltd) |
| **40583** | Dermovate-NN cream (Chemidex Pharma Ltd) |
| **40698** | Dermovate-NN ointment (Chemidex Pharma Ltd) |
| **949** | Eumovate 0.05% ointment (GlaxoSmithKline UK Ltd) |
| **1562** | Clobetasone 0.05% cream |
| **50485** | Eumovate 0.05% cream (Sigma Pharmaceuticals Plc) |
| **1005** | Eumovate 0.05% cream (GlaxoSmithKline UK Ltd) |
| **4837** | Clobetasone 0.05% ointment |
| **61533** | Clobetasone 0.05% ointment (A A H Pharmaceuticals Ltd) |
| **59985** | Clobavate 0.05% ointment (Auden McKenzie (Pharma Division) Ltd) |
| **16862** | Eumovate Eczema and Dermatitis 0.05% cream (GlaxoSmithKline Consumer Healthcare) |
| **52789** | Eumovate 0.05% cream (Waymade Healthcare Plc) |
| **17838** | Cloburate Eye drops (Dominion Pharma) |
| **17290** | Clobetasone butyrate 0.1% eye drops |
| **1389** | Trimovate cream (GlaxoSmithKline UK Ltd) |
| **56464** | Generic Trimovate cream |
| **42505** | Betamethasone valerate with clotrimazole cream |
| **1265** | Clotrimazole 1% with Hydrocortisone 1% cream |
| **3575** | Lotriderm Cream (Dominion Pharma) |
| **5217** | Betamethasone dipropionate 0.064% / Clotrimazole 1% cream |
| **5905** | Lotriderm cream (Teva UK Ltd) |
| **1009** | Canesten HC cream (Bayer Plc) |
| **5446** | Canesten Hydrocortisone cream (Bayer Plc) |
| **49193** | Canesten HC cream (DE Pharmaceuticals) |
| **55937** | Canesten HC cream (Waymade Healthcare Plc) |
| **9724** | Hydrocortisone 1% / Clotrimazole 1% cream |
| **1239** | Alphosyl hc Cream (Stafford-Miller Ltd) |
| **11527** | Coal tar with allantoin with hydrocortisone cream |
| **55386** | Coal tar solution 5% in Betamethasone valerate 0.025% ointment |
| **58785** | Coal tar solution 5% in Clobetasol 0.05% ointment |
| **61858** | Coal tar solution 5% in Clobetasone 0.05% cream |
| **55748** | Coal tar 5% in Clobetasone 0.05% ointment |
| **7548** | Cortisone 5mg capsules |
| **229** | Cortisone 25mg tablets |
| **53143** | Cortisone 25mg tablets (A A H Pharmaceuticals Ltd) |
| **23210** | Cortistab 5mg Tablet (Waymade Healthcare Plc) |
| **12400** | Cortisyl 25mg Tablet (Aventis Pharma) |
| **12398** | Cortelan 25mg Tablet (Glaxo Laboratories Ltd) |
| **10574** | Cortisone acetate 5mg tablets |
| **18637** | Cortistab 25mg Tablet (Waymade Healthcare Plc) |
| **53705** | Cortisone acetate 5mg Capsule (Martindale Pharmaceuticals Ltd) |
| **4113** | Crotamiton 10% with Hydrocortisone 0.25% cream |
| **20577** | Calcort 6mg Tablet (Shire Pharmaceuticals Ltd) |
| **9375** | Deflazacort 1mg tablets |
| **3992** | Deflazacort 6mg tablets |
| **22555** | Calcort 1mg tablets (Shire Pharmaceuticals Ltd) |
| **41335** | Calcort 6mg tablets (Sanofi) |
| **34207** | Tridesilon 0.05% Cream (Lagap) |
| **10943** | Stiedex 0.25% oily Cream (Stiefel Laboratories (UK) Ltd) |
| **12410** | Desoximetasone 0.25% oily cream |
| **8854** | Stiedex LP 0.05% oily cream (Stiefel Laboratories (UK) Ltd) |
| **6341** | Desoximetasone 0.05% oily cream |
| **56463** | Maxidex 0.1% eye drops (Waymade Healthcare Plc) |
| **47238** | Dexamethasone 700microgram intravitreal implant with device |
| **57850** | Maxidex 0.1% eye drops (Dowelhurst Ltd) |
| **38105** | Dexamethasone eye drops preservative free |
| **47319** | Ozurdex 700microgram intravitreal implant in applicator (Allergan Ltd) |
| **1106** | Maxidex 0.1% eye drops (Alcon Laboratories (UK) Ltd) |
| **43439** | Dexamethasone 0.1% eye ointment |
| **51864** | Maxidex 0.1% eye drops (Lexon (UK) Ltd) |
| **53463** | Maxidex 0.1% eye drops (Mawdsley-Brooks & Company Ltd) |
| **1127** | Dexamethasone 0.1% eye drops |
| **50594** | Dexamethasone 0.1% eye drops preservative free |
| **17374** | Framycetin with dexamethasone and gramicidin Eye gel |
| **14606** | Framycetin with dexamethasone and gramicidin Eye gel |
| **7289** | Sofradex Ear/eye drops solution (Distriphar (UK)) |
| **12793** | Dexamethasone with framycetin with gramicidin ear ointment |
| **27073** | Framycetin with dexamethasone and gramicidin Ear/eye drops solution |
| **13257** | Dexamethasone with framycetin with gramicidin eye drops |
| **4794** | Framycetin with dexamethasone and gramicidin Ear drops |
| **6027** | Dexamethasone with framycetin with gramicidin ear drops |
| **309** | Sofradex Ear drops (Sanofi) |
| **14665** | Dexamethasone with framycetin with gramicidin eye ointment |
| **34137** | Sofradex Ear/eye drops solution (Distriphar (UK)) |
| **1846** | Sofradex Eye ointment (Distriphar (UK)) |
| **14565** | Framycetin with dexamethasone and gramicidin Eye drops |
| **7360** | Dexamethasone with framycetin with gramicidin ear/eye drops |
| **4627** | Sofradex Eye drops (Distriphar (UK)) |
| **4916** | Sofradex Ear ointment (Distriphar (UK)) |
| **9994** | Decadron 500microgram tablets (Merck Sharp & Dohme Ltd) |
| **1280** | Dexamethasone 2mg tablets |
| **34880** | Dexamethasone 2mg tablets (Aspen Pharma Trading Ltd) |
| **4779** | Dexamethasone 500microgram tablets |
| **55401** | Dexamethasone 500microgram tablets (A A H Pharmaceuticals Ltd) |
| **34915** | Dexamethasone 500microgram tablets (Organon Laboratories Ltd) |
| **60120** | Dexamethasone 2mg tablets (Alliance Healthcare (Distribution) Ltd) |
| **5157** | Dexamethasone 2mg/5ml oral solution |
| **21903** | Oradexon-organon 2mg Tablet (Organon Laboratories Ltd) |
| **34801** | Dexamethasone 0.5mg/5ml Oral solution (Rosemont Pharmaceuticals Ltd) |
| **36055** | Dexamethasone 2mg Tablet (Hillcross Pharmaceuticals Ltd) |
| **53207** | Dexamethasone tablets |
| **56347** | Dexamethasone 5mg/5ml oral solution |
| **45234** | Dexamethasone 100microgram capsules |
| **186** | Dexamethasone 500micrograms/5ml oral solution |
| **54793** | Dexamethasone 2mg/5ml oral suspension |
| **52396** | Dexamethasone 1mg/5ml oral solution |
| **39845** | Sofradex ear/eye drops (Sanofi) |
| **56225** | Generic Sofradex ear/eye drops |
| **7067** | Dexamethasone 0.1% eye drops 0.5ml unit dose preservative free |
| **59044** | Dropodex 0.1% eye drops 0.4ml unit dose (Moorfields Pharmaceuticals) |
| **5237** | Minims dexamethasone 0.1% eye drops 0.5ml unit dose (Bausch & Lomb UK Ltd) |
| **35453** | Dexamethasone 3.3mg/1ml solution for injection ampoules |
| **13972** | Dexamethasone sodium phosphate 5mg/ml injection |
| **37500** | Dexamethasone 6.6mg/2ml solution for injection vials |
| **26454** | Decadron 4mg/ml Injection (MSD Thomas Morson Pharmaceuticals) |
| **31948** | Dexamethasone 4mg/ml Injection (Mayne Pharma Plc 1) |
| **4233** | Dexamethasone sodium phosphate 4mg/ml injection |
| **14906** | Dexamethasone 4mg/1ml solution for injection ampoules |
| **26299** | Oradexon-organon 4mg/ml Intraarticular injection (Organon Laboratories Ltd) |
| **61316** | Dexamethasone 4mg/1ml solution for injection ampoules (Alliance Healthcare (Distribution) Ltd) |
| **53173** | Dexamethasone 4mg/1ml solution for injection ampoules (Aspen Pharma Trading Ltd) |
| **13952** | Decadron 4mg/ml Injection (MSD Thomas Morson Pharmaceuticals) |
| **10657** | Dexamethasone sodium phosphate 4mg/ml intra-artic injection |
| **11334** | Dexamethasone sodium phosphate iv 4mg/ml injection |
| **34083** | Dexamethasone 5mg/ml Injection (Organon Laboratories Ltd) |
| **26300** | Dexamethasone 20mg/ml shock pack |
| **19259** | Dexamethasone 8mg/2ml solution for injection vials |
| **47598** | Dexamethasone 8mg/2ml solution for injection vials (Organon Laboratories Ltd) |
| **21668** | Decadron shock pak 20 20mg/ml Shock pack (Merck Sharp & Dohme Ltd) |
| **28215** | Dexamethasone 100mg/5ml solution for injection vials |
| **21218** | Dexsol 2mg/5ml oral solution (Rosemont Pharmaceuticals Ltd) |
| **58474** | Dexamethasone 2mg/5ml oral solution sugar free (A A H Pharmaceuticals Ltd) |
| **4943** | Dexamethasone 2mg/5ml oral solution sugar free |
| **49712** | Sofradex ear/eye ointment (Florizel Ltd) |
| **1895** | Maxitrol eye drops (Alcon Laboratories (UK) Ltd) |
| **48896** | Generic Maxitrol eye drops |
| **7032** | Maxitrol eye ointment (Alcon Laboratories (UK) Ltd) |
| **53160** | Generic Maxitrol eye ointment |
| **3143** | Nerisone Forte 0.3% oily cream (Meadow Laboratories Ltd) |
| **12626** | Nerisone Forte 0.3% ointment (Meadow Laboratories Ltd) |
| **10621** | Diflucortolone 0.1% ointment |
| **6176** | Diflucortolone 0.1% cream |
| **1228** | Nerisone 0.1% oily cream (Meadow Laboratories Ltd) |
| **12727** | Diflucortolone 0.3% ointment |
| **2831** | Nerisone 0.1% cream (Meadow Laboratories Ltd) |
| **6096** | Diflucortolone 0.3% oily cream |
| **3123** | Diflucortolone 0.1% oily cream |
| **34117** | Temetex Ointment (Roche Products Ltd) |
| **4405** | Nerisone 0.1% ointment (Meadow Laboratories Ltd) |
| **4158** | Pevaryl TC Cream (Lorex Synthelabo Ltd) |
| **17373** | Econazole with triamcinolone cream |
| **8089** | Econazole with hydrocortisone 1% with 1% cream |
| **2576** | Econacort cream (Bristol-Myers Squibb Pharmaceuticals Ltd) |
| **5699** | Econazole 1% / Hydrocortisone 1% cream |
| **28359** | Fluclorolone acetonide 0.025%w/w Ointment |
| **8627** | Fluclorolone acetonide 0.025%w/w Cream |
| **4251** | Topilar 0.025%w/w Cream (Bioglan Laboratories Ltd) |
| **12473** | Topilar 0.025%w/w Ointment (Bioglan Laboratories Ltd) |
| **60516** | Fludrocortisone 50microgram capsules |
| **37622** | Fludrocortisone Capsule |
| **2439** | Fludrocortisone 100microgram tablets |
| **52472** | Fludrocortisone 50micrograms/5ml oral suspension |
| **2434** | Florinef 100microgram tablets (Bristol-Myers Squibb Pharmaceuticals Ltd) |
| **37870** | Fludrocortisone Liquid |
| **7607** | Haelan -c Cream (Dista Products Ltd) |
| **7723** | Fludroxycortide with clioquinol Cream |
| **8269** | Haelan -c Ointment (Dista Products Ltd) |
| **18322** | Fludroxycortide with clioquinol Ointment |
| **47359** | Clioquinol with Fludroxycortide cream |
| **24849** | Fludroxycortide 0.05% Ointment |
| **21448** | Haelan -x 0.05% Cream (Dista Products Ltd) |
| **12351** | Fludroxycortide 0.05% Cream |
| **18691** | Haelan -x 0.05% Ointment (Dista Products Ltd) |
| **3124** | Fludroxycortide 0.0125% cream |
| **3849** | Haelan 0.0125% cream (Typharm Ltd) |
| **3662** | Haelan 0.0125% ointment (Typharm Ltd) |
| **5272** | Haelan 4micrograms/square cm tape 7.5cm (Typharm Ltd) |
| **7370** | Fludroxycortide 4micrograms/square cm tape 7.5cm |
| **8319** | Fludroxycortide 0.0125% ointment |
| **6086** | Syntaris 0.025% nasal spray (Teva UK Ltd) |
| **4299** | Flunisolide 25micrograms/dose nasal spray |
| **2150** | Syntaris 25microgram/actuation Nasal spray (Roche Products Ltd) |
| **12589** | Syntaris hayfever 25microgram/actuation Nasal spray (Roche Consumer Health) |
| **34161** | Fluocinolone acetonide 0.02% Cream |
| **47811** | Synalar Forte Cream (Ici Pharmaceuticals) |
| **1772** | Synalar Cream (GP Pharma) |
| **7844** | Fluocinolone acetonide 0.1%w/w Ointment |
| **12366** | Fluocinolone acetonide 0.1%w/w Cream |
| **2461** | Synalar Ointment (GP Pharma) |
| **7718** | Fluocinolone acetonide 0.025% ointment |
| **5317** | Synalar 1 in 10 Dilution 0.0025% cream (Derma UK Ltd) |
| **41379** | Synalar 0.025% ointment (Derma UK Ltd) |
| **41380** | Synalar 0.025% cream (Derma UK Ltd) |
| **11464** | Fluocinolone acetonide 0.00625% ointment |
| **10982** | Fluocinolone acetonide 0.00625% cream |
| **5533** | Fluocinolone acetonide 0.025% cream |
| **4773** | Fluocinolone acetonide 0.025% gel |
| **3082** | Synalar 1 in 4 Dilution 0.00625% cream (Derma UK Ltd) |
| **7843** | Synalar 1 in 4 Dilution 0.00625% ointment (Derma UK Ltd) |
| **11425** | Fluocinolone acetonide 0.0025% cream |
| **1386** | Synalar 0.025% gel (Derma UK Ltd) |
| **59821** | Tri-Luma cream (Imported (United States)) |
| **8270** | Metosyn 0.05% Scalp lotion (GP Pharma) |
| **2958** | Metosyn Ointment (GP Pharma) |
| **2550** | Metosyn fapg Cream (GP Pharma) |
| **10411** | Fluocinonide 0.05% Scalp lotion |
| **41767** | Metosyn FAPG 0.05% cream (Derma UK Ltd) |
| **4281** | Fluocinonide 0.05% cream |
| **41886** | Metosyn 0.05% ointment (Derma UK Ltd) |
| **1523** | Fluocinonide 0.05% ointment |
| **3589** | Ultradil plain Ointment (Schering Health Care Ltd) |
| **10824** | Ultralanum Plain cream (Meadow Laboratories Ltd) |
| **13249** | Fluocortolone hexanoate 0.25% / Fluocortolone pivalate 0.25% cream |
| **3592** | Ultradil plain Cream (Schering Health Care Ltd) |
| **22807** | Fluocortolone 0.1% Cream |
| **19403** | Fluocortolone with cinchocaine Suppository |
| **11273** | Fluocortolone with cinchocaine Ointment |
| **36530** | Ultralanum Ointment (Schering Health Care Ltd) |
| **4486** | Ultralanum Plain ointment (Meadow Laboratories Ltd) |
| **18393** | Fluocortolone 0.25% / Fluocortolone hexanoate 0.25% ointment |
| **24876** | Fluorometholone with neomycin Eye drops |
| **8963** | Fml-neo Eye drops (Allergan Ltd) |
| **3866** | FML Liquifilm 0.1% ophthalmic suspension (Allergan Ltd) |
| **4678** | Fluorometholone 0.1% eye drops |
| **39529** | Avamys 27.5micrograms/dose nasal spray (GlaxoSmithKline UK Ltd) |
| **39455** | Fluticasone furoate 27.5micrograms/dose nasal spray |
| **1835** | Cutivate 0.05% cream (GlaxoSmithKline UK Ltd) |
| **4117** | Fluticasone 0.005% ointment |
| **5033** | Fluticasone 0.05% cream |
| **4107** | Cutivate 0.005% ointment (GlaxoSmithKline UK Ltd) |
| **39733** | Fluticasone propionate 50micrograms/dose nasal spray (A A H Pharmaceuticals Ltd) |
| **5536** | Flixonase Nasule 400microgram/unit dose nasal drops (GlaxoSmithKline UK Ltd) |
| **45517** | Nasofan Allergy 50micrograms/dose nasal spray (Teva UK Ltd) |
| **2336** | Fluticasone propionate 50micrograms/dose nasal spray |
| **57010** | Fluticasone propionate 50micrograms/dose nasal spray (Sigma Pharmaceuticals Plc) |
| **22357** | Nasofan 50micrograms/dose aqueous nasal spray (Teva UK Ltd) |
| **943** | Flixonase 50micrograms/dose aqueous nasal spray (GlaxoSmithKline UK Ltd) |
| **50743** | Flixonase Nasule 400microgram/unit dose nasal drops (Sigma Pharmaceuticals Plc) |
| **4850** | Fluticasone 400microgram/unit dose nasal drops |
| **60438** | Flixonase 50micrograms/dose aqueous nasal spray (DE Pharmaceuticals) |
| **45908** | Pirinase Hayfever 0.05% nasal spray (GlaxoSmithKline Consumer Healthcare) |
| **50398** | Flixonase Nasule 400microgram/unit dose nasal drops (Mawdsley-Brooks & Company Ltd) |
| **11772** | Flixonase allergy 50 microgram/spray Spray (GlaxoSmithKline Consumer Healthcare) |
| **37447** | Fluticasone propionate 50microgram inhalation powder blisters |
| **1518** | Flixotide 50microgram/actuation Inhalation powder (Allen & Hanburys Ltd) |
| **4131** | Fluticasone 100microgram Disc |
| **7638** | Fluticasone 250microgram Disc |
| **4688** | Fluticasone 50microgram/actuation Pressurised inhalation |
| **7948** | Fluticasone propionate 250micrograms/dose dry powder inhaler |
| **5975** | Fluticasone 125micrograms/dose inhaler CFC free |
| **36021** | Fluticasone propionate 50microgram inhalation powder blisters with device |
| **911** | Flixotide accuhaler 250 250microgram/inhalation Inhalation powder (Allen & Hanburys Ltd) |
| **9164** | Fluticasone propionate 50micrograms/dose dry powder inhaler |
| **42994** | Flixotide 250micrograms/dose Accuhaler (GlaxoSmithKline UK Ltd) |
| **42928** | Flixotide 100micrograms/dose Accuhaler (GlaxoSmithKline UK Ltd) |
| **35905** | Fluticasone propionate 250microgram inhalation powder blisters |
| **35986** | Flixotide 50microgram disks (GlaxoSmithKline UK Ltd) |
| **4132** | Fluticasone 125microgram/actuation Pressurised inhalation |
| **42985** | Flixotide 50micrograms/dose Accuhaler (GlaxoSmithKline UK Ltd) |
| **35392** | Flixotide 500microgram disks with Diskhaler (GlaxoSmithKline UK Ltd) |
| **35374** | Flixotide 500microgram disks (GlaxoSmithKline UK Ltd) |
| **5718** | Flixotide 125micrograms/dose Evohaler (GlaxoSmithKline UK Ltd) |
| **36290** | Flixotide 50microgram disks with Diskhaler (GlaxoSmithKline UK Ltd) |
| **2282** | Fluticasone propionate 500micrograms/dose dry powder inhaler |
| **56484** | Flixotide 250micrograms/dose Accuhaler (Waymade Healthcare Plc) |
| **7602** | Fluticasone 50microgram Disc |
| **11478** | Fluticasone 2mg/2ml nebuliser liquid unit dose vials |
| **36462** | Fluticasone propionate 500microgram inhalation powder blisters |
| **5885** | Fluticasone propionate 100micrograms/dose dry powder inhaler |
| **53057** | Flixotide 50micrograms/dose Evohaler (Lexon (UK) Ltd) |
| **57525** | Flixotide 250micrograms/dose Accuhaler (Stephar (U.K.) Ltd) |
| **1424** | Flixotide 250microgram Disc (Allen & Hanburys Ltd) |
| **56499** | Flixotide 500micrograms/dose Accuhaler (Waymade Healthcare Plc) |
| **5309** | Flixotide 50micrograms/dose Evohaler (GlaxoSmithKline UK Ltd) |
| **35700** | Fluticasone propionate 500microgram inhalation powder blisters with device |
| **1426** | Flixotide 500microgram Disc (Allen & Hanburys Ltd) |
| **57555** | Flixotide 125micrograms/dose Evohaler (Dowelhurst Ltd) |
| **2951** | Fluticasone 250microgram/actuation Pressurised inhalation |
| **8635** | Flixotide 50microgram Disc (Allen & Hanburys Ltd) |
| **1676** | Flixotide 125microgram/actuation Inhalation powder (Allen & Hanburys Ltd) |
| **49772** | Fluticasone 250micrograms/dose Evohaler (Sigma Pharmaceuticals Plc) |
| **1412** | Flixotide 250microgram/actuation Inhalation powder (Allen & Hanburys Ltd) |
| **43074** | Flixotide 500micrograms/dose Accuhaler (GlaxoSmithKline UK Ltd) |
| **56474** | Flixotide 125micrograms/dose Evohaler (DE Pharmaceuticals) |
| **35638** | Fluticasone propionate 100microgram inhalation powder blisters with device |
| **17465** | Fluticasone 500micrograms/2ml nebuliser liquid unit dose vials |
| **36090** | Flixotide 100microgram disks (GlaxoSmithKline UK Ltd) |
| **5223** | Fluticasone 50micrograms/dose inhaler CFC free |
| **3989** | Flixotide 100microgram Disc (Allen & Hanburys Ltd) |
| **3289** | Flixotide 25micrograms/dose inhaler (GlaxoSmithKline UK Ltd) |
| **5683** | Flixotide 250micrograms/dose Evohaler (GlaxoSmithKline UK Ltd) |
| **57579** | Flixotide 50micrograms/dose Accuhaler (DE Pharmaceuticals) |
| **4926** | Flixotide accuhaler 100 100microgram/inhalation Inhalation powder (Allen & Hanburys Ltd) |
| **35611** | Flixotide 250microgram disks (GlaxoSmithKline UK Ltd) |
| **16305** | Flixotide 2mg/2ml Nebules (GlaxoSmithKline UK Ltd) |
| **5822** | Fluticasone 250micrograms/dose inhaler CFC free |
| **35772** | Fluticasone propionate 100microgram inhalation powder blisters |
| **2440** | Flixotide accuhaler 500 500microgram/inhalation Inhalation powder (Allen & Hanburys Ltd) |
| **5580** | Flixotide accuhaler 50 50microgram/inhalation Inhalation powder (Allen & Hanburys Ltd) |
| **56475** | Flixotide 50micrograms/dose Accuhaler (Sigma Pharmaceuticals Plc) |
| **35225** | Flixotide 100microgram disks with Diskhaler (GlaxoSmithKline UK Ltd) |
| **56477** | Flixotide 100micrograms/dose Accuhaler (Waymade Healthcare Plc) |
| **5551** | Flixotide 0.5mg/2ml Nebules (GlaxoSmithKline UK Ltd) |
| **51815** | Flixotide 250micrograms/dose Evohaler (Waymade Healthcare Plc) |
| **2723** | Fluticasone 25micrograms/dose inhaler |
| **36401** | Fluticasone propionate 250microgram inhalation powder blisters with device |
| **35461** | Flixotide 250microgram disks with Diskhaler (GlaxoSmithKline UK Ltd) |
| **7891** | Fluticasone 500microgram Disc |
| **11588** | Fluticasone 125micrograms/dose / Salmeterol 25micrograms/dose inhaler CFC free |
| **48739** | Seretide 250 Evohaler (DE Pharmaceuticals) |
| **53230** | Seretide 250 Accuhaler (DE Pharmaceuticals) |
| **51151** | Seretide 125 Evohaler (Lexon (UK) Ltd) |
| **61280** | Seretide 250 Accuhaler (Waymade Healthcare Plc) |
| **5143** | Seretide 50 Evohaler (GlaxoSmithKline UK Ltd) |
| **50886** | Seretide 250 Evohaler (Stephar (U.K.) Ltd) |
| **5161** | Seretide 125 Evohaler (GlaxoSmithKline UK Ltd) |
| **13040** | Fluticasone propionate 250micrograms/dose / Salmeterol 50micrograms/dose dry powder inhaler |
| **638** | Seretide 250 Accuhaler (GlaxoSmithKline UK Ltd) |
| **51909** | Seretide 250 Evohaler (Necessity Supplies Ltd) |
| **5172** | Seretide 250 Evohaler (GlaxoSmithKline UK Ltd) |
| **11618** | Fluticasone 250micrograms/dose / Salmeterol 25micrograms/dose inhaler CFC free |
| **49000** | Seretide 250 Evohaler (Waymade Healthcare Plc) |
| **12994** | Fluticasone 50micrograms/dose / Salmeterol 25micrograms/dose inhaler CFC free |
| **51027** | Seretide 125 Evohaler (DE Pharmaceuticals) |
| **50560** | Seretide 250 Accuhaler (Sigma Pharmaceuticals Plc) |
| **50689** | Flutiform 50micrograms/dose / 5micrograms/dose inhaler (Napp Pharmaceuticals Ltd) |
| **51270** | Fluticasone 50micrograms/dose / Formoterol 5micrograms/dose inhaler CFC free |
| **4840** | Fusidic acid with betamethasone valerate 2%with1% cream |
| **3300** | Genticin with hydrocortisone Cream (Nicholas Laboratories Ltd) |
| **3655** | Gentamicin with hydrocortisone Ointment |
| **3103** | Genticin with hydrocortisone Ointment (Nicholas Laboratories Ltd) |
| **13929** | Hydrocortisone powder |
| **1362** | Tarcortin Cream (Stafford-Miller Ltd) |
| **7982** | Carbo-cort Cream (Lagap) |
| **8092** | Hydrocortisone and coal tar Cream |
| **8006** | Coal tar with hydrocortisone cream |
| **33649** | Hydrocortisone 1% Cream (Teva UK Ltd) |
| **34906** | Hydrocortisone 1% Ointment (Celltech Pharma Europe Ltd) |
| **2190** | Hydrocortisone 0.125% Cream |
| **10518** | Hydrocortisone with dimeticone 0.5% with 20% Cream |
| **39737** | Hydrocortisone 0.50% Ointment (C P Pharmaceuticals Ltd) |
| **47809** | Hydrocortisone 1% Ointment (Thornton & Ross Ltd) |
| **4373** | Cobadex 1% Cream (Actavis UK Ltd) |
| **3859** | Hydrocortisyl 1%w/w Cream (Hoechst Marion Roussel) |
| **14555** | Hydrocortisone with dimeticone 1% with 20% Cream |
| **10751** | Hydrocortistab 1% Ointment (Waymade Healthcare Plc) |
| **32377** | Lexon hydrocortisone 1% Cream (Lexon (UK) Ltd) |
| **7892** | Hydrocortisone 1% Cream |
| **19187** | Hydrocortisone 1% Ointment (Teva UK Ltd) |
| **34326** | Hydrocortisone 0.50% Ointment (Teva UK Ltd) |
| **31806** | Jungle formula bite and sting relief 1% Cream (Chefaro UK Ltd) |
| **41406** | Hydrocortisone 2.50% Cream (Martindale Pharmaceuticals Ltd) |
| **3567** | Hydrocortistab 1% Cream (Waymade Healthcare Plc) |
| **10517** | Cobadex 0.50% Cream (Actavis UK Ltd) |
| **34454** | Hydrocortisone 0.50% Cream (Teva UK Ltd) |
| **34900** | Hydrocortisone 1% Cream (Celltech Pharma Europe Ltd) |
| **8764** | Hydrocortisyl 1%w/w Ointment (Hoechst Marion Roussel) |
| **59914** | Derma Care Hydrocortisone 1% cream (The Boots Company Plc) |
| **47801** | Hydrocortisone Bite and Sting Relief 1% cream (Galpharm International Ltd) |
| **24513** | Zenoxone 1% cream (Teva UK Ltd) |
| **34846** | Hydrocortisone 0.5% ointment (A A H Pharmaceuticals Ltd) |
| **734** | Hydrocortisone 1% ointment |
| **4214** | Efcortelan 0.5% ointment (Chemidex Pharma Ltd) |
| **34317** | Hydrocortisone 1% cream (Actavis UK Ltd) |
| **55572** | Hydrocortisone 1% cream (Sovereign Medical Ltd) |
| **945** | Hydrocortisone 0.5% ointment |
| **33655** | Hydrocortisone 0.5% ointment (Actavis UK Ltd) |
| **33081** | Hydrocortisone 2.5% ointment (A A H Pharmaceuticals Ltd) |
| **8986** | Efcortelan 2.5% ointment (Chemidex Pharma Ltd) |
| **34291** | Hydrocortisone 1% cream (A A H Pharmaceuticals Ltd) |
| **982** | Hydrocortisone 0.5% cream |
| **2844** | Hydrocortisone 2.5% ointment |
| **2075** | Dioderm 0.1% cream (Dermal Laboratories Ltd) |
| **39818** | Hydrocortisone 0.5% cream (Actavis UK Ltd) |
| **3561** | Efcortelan 1% cream (Chemidex Pharma Ltd) |
| **46091** | Hydrocortisone 2.5% ointment (Essential Generics Ltd) |
| **8479** | Efcortelan 1% ointment (Chemidex Pharma Ltd) |
| **40077** | Exe-Cort Hydrocortisone 1% cream (Nucare Plc) |
| **8569** | Mildison Lipocream 1% cream (Astellas Pharma Ltd) |
| **310** | Hydrocortisone 0.1% cream |
| **34767** | Hydrocortisone 2.5% cream (Teva UK Ltd) |
| **48092** | Hydrocortisone 1% cream (Essential Generics Ltd) |
| **73** | Hydrocortisone 1% cream |
| **47845** | Hydrocortisone 1% cream (Teva UK Ltd) |
| **32098** | Hydrocortisone 1% ointment (Teva UK Ltd) |
| **8313** | Efcortelan 2.5% cream (GlaxoSmithKline UK Ltd) |
| **34459** | Hydrocortisone 1% ointment (A A H Pharmaceuticals Ltd) |
| **40402** | Hydrocortisone 2.5% cream (A A H Pharmaceuticals Ltd) |
| **3166** | Efcortelan 0.5% cream (Chemidex Pharma Ltd) |
| **43506** | Hydrocortisone 1% cream (Pinewood Healthcare) |
| **34627** | Hydrocortisone 1% cream (Vantage) |
| **57818** | Hydrocortisone 2.5% cream (Essential Generics Ltd) |
| **32096** | Hydrocortisone 1% ointment (Actavis UK Ltd) |
| **34896** | Hydrocortisone 0.5% cream (A A H Pharmaceuticals Ltd) |
| **17799** | Dermacort hydrocortisone 0.1% cream (Marlborough Pharmaceuticals Ltd) |
| **32877** | Hydrocortisone 2.5% ointment (Teva UK Ltd) |
| **1061** | Hydrocortisone 2.5% cream |
| **57931** | Hydrocortisone 20mg tablets (Teva UK Ltd) |
| **38022** | Hydrocortisone 10mg/5ml oral suspension |
| **51872** | Hydrocortisone 2.5mg capsules |
| **6098** | Hydrocortone 10mg tablets (Auden McKenzie (Pharma Division) Ltd) |
| **4535** | Hydrocortisone 20mg tablets |
| **51824** | Hydrocortisone 5mg/5ml oral suspension sugar free |
| **3418** | Hydrocortisone 10mg tablets |
| **13043** | Hydrocortone 20mg tablets (Auden McKenzie (Pharma Division) Ltd) |
| **51722** | Hydrocortisone 5mg/5ml oral suspension |
| **38054** | Hydrocortisone Tablet |
| **10754** | Hydrocortistab 20mg Tablet (Waymade Healthcare Plc) |
| **14076** | Hydrocortisone 5mg/5ml Oral solution |
| **58047** | Hydrocortisone 100mg suppositories |
| **22434** | Hydrocortisone acetate Powder |
| **52153** | Hydrocortisone acetate Powder (Biorex Laboratories Ltd) |
| **5244** | Fusidic acid with hydrocortisone 2%with1% cream |
| **12801** | Fusidic acid with hydrocortisone 2%with1% gel |
| **42501** | Hydrocortisone with fusidic acid Ointment |
| **20460** | Hydrocortisone with sodium fusidate 1%with2% Ointment |
| **12611** | Hydrocortisone with fusidic acid 1% with 2% gel |
| **1944** | Fucidin h Gel (LEO Pharma) |
| **10451** | Framycort Ointment (Rpr / Fisons) |
| **43526** | Hydrocortisone 0.5% eye ointment (Martindale Pharmaceuticals Ltd) |
| **9471** | Hydrocortisone 0.5% eye ointment |
| **14986** | Hydrocortisone acetate 1% cream |
| **27686** | Lanacort 1% cream (Reckitt Benckiser Healthcare (UK) Ltd) |
| **9274** | Hc45 Hydrocortisone 1% cream (Reckitt Benckiser Healthcare (UK) Ltd) |
| **28589** | Hydrocortisone acetate 1% ointment |
| **55847** | Hydrocortisone 1% Eye ointment (C P Pharmaceuticals Ltd) |
| **12267** | Framycetin with hydrocortisone Eye drops |
| **17839** | Framycetin with hydrocortisone Eye ointment |
| **2692** | Framycort Eye drops (Rpr / Fisons) |
| **41686** | Hydrocortisone 0.50% Eye ointment (C P Pharmaceuticals Ltd) |
| **3643** | Hydrocortisone 1% eye ointment |
| **8108** | Hydrocortisone acetate 25mg/1ml suspension for injection ampoules |
| **1893** | Hydrocortistab 25mg/1ml suspension for injection ampoules (AMCo) |
| **21687** | Hydrocortisone acetate and pramocaine hc 1% with 1% Cream |
| **9428** | Proctocream HC 1%+1% Cream (Stafford-Miller Ltd) |
| **1432** | Xyloproct Suppository (AstraZeneca UK Ltd) |
| **9324** | Lidocaine with hydrocortisone acetate 5% + 0.275% Suppository |
| **1567** | Colifoam 10% aerosol (Meda Pharmaceuticals Ltd) |
| **292** | Hydrocortisone 10% foam aerosol enema |
| **10677** | Hydrocortisone retention enema |
| **52969** | Colifoam 10% aerosol (Mawdsley-Brooks & Company Ltd) |
| **14605** | Hydrocortisone 2.5% eye ointment |
| **8475** | Hydrocortisone 1% eye drops |
| **6170** | Anugesic-HC suppositories (Pfizer Ltd) |
| **242** | Fucidin H cream (LEO Pharma) |
| **4638** | Hydrocortisone acetate 1% / Fusidic acid 2% cream |
| **668** | Gentamicin 0.3% / Hydrocortisone acetate 1% ear drops |
| **1111** | Gentisone HC ear drops (AMCo) |
| **10512** | Hydrocortisone 25mg Suppository |
| **38109** | Hydrocortisone 25mg Suppository (Celltech Pharma Europe Ltd) |
| **14811** | Neo-cortef Eye drops (PLIVA Pharma Ltd) |
| **3292** | Neo-cortef Eye drops (Dominion Pharma) |
| **16812** | Hydrocortisone 1% / Neomycin 0.5% cream |
| **57459** | Hydrocortisone 1.5% / Neomycin 0.5% ear/eye ointment |
| **9494** | Hydrocortisone 1.5% / Neomycin 0.5% ear/eye drops |
| **51684** | Neo-Cortef ear/eye ointment (PLIVA Pharma Ltd) |
| **52083** | Neo-Cortef ear/eye drops (PLIVA Pharma Ltd) |
| **6969** | Proctofoam HC foam enema (Meda Pharmaceuticals Ltd) |
| **8852** | Hydrocortisone 1% / Pramocaine 1% foam enema |
| **8336** | Epifoam Foam (Stafford-Miller Ltd) |
| **4348** | Foam (Stafford-Miller Ltd) |
| **946** | Fucidin H ointment (LEO Pharma) |
| **2875** | Hydrocortisone acetate 1% / Sodium fusidate 2% ointment |
| **6385** | Hydrocortisone butyrate 0.1% scalp lotion |
| **4599** | Hydrocortisone butyrate 0.1% ointment |
| **9811** | Locoid Crelo 0.1% topical emulsion (Astellas Pharma Ltd) |
| **2914** | Locoid 0.1% ointment (Astellas Pharma Ltd) |
| **4428** | Locoid 0.1% scalp lotion (Astellas Pharma Ltd) |
| **2103** | Hydrocortisone butyrate 0.1% cream |
| **15918** | Hydrocortisone 0.1% topical emulsion |
| **2102** | Locoid 0.1% cream (Astellas Pharma Ltd) |
| **2853** | Locoid 0.1% Lipocream (Astellas Pharma Ltd) |
| **51371** | Locoid 0.1% scalp lotion (Mawdsley-Brooks & Company Ltd) |
| **37638** | Efcortesol 500mg/5ml solution for injection ampoules (AMCo) |
| **35172** | Efcortesol 100mg/1ml solution for injection ampoules (AMCo) |
| **43355** | Hydrocortisone sodium phosphate 500mg/5ml solution for injection ampoules |
| **35175** | Hydrocortisone sodium phosphate 100mg/1ml solution for injection ampoules |
| **9574** | Hydrocortisone na phosphate 100mg/ml Injection |
| **2615** | Efcortesol 100mg/ml Injection (Sovereign Medical Ltd) |
| **46280** | Hydrocortisone 2.5mg muco-adhesive buccal tablets sugar free (Auden McKenzie (Pharma Division) Ltd) |
| **1332** | Corlan 2.5mg Pellets (Auden McKenzie (Pharma Division) Ltd) |
| **6339** | Hydrocortisone 2.5mg muco-adhesive buccal tablets sugar free |
| **56319** | Hydrocortisone 2.5mg muco-adhesive buccal tablets sugar free (A A H Pharmaceuticals Ltd) |
| **5118** | Hydrocortisone na succinate 2.5mg Lozenge |
| **61791** | Hydrocortisone 2.5mg muco-adhesive buccal tablets sugar free (Waymade Healthcare Plc) |
| **2338** | Chloromycetin with hydrocortisone 1%+0.5% Eye ointment (Parke-davis Research Laboratories) |
| **1777** | Chloramphenicol with Hydrocortisone eye ointment |
| **51167** | Solu-Cortef 100mg powder and solvent for solution for injection vials (Pfizer Ltd) |
| **49498** | Hydrocortisone sodium succinate 100mg powder for solution for injection vials |
| **54715** | Solu-Cortef 100mg powder for solution for injection vials (Pfizer Ltd) |
| **49707** | Hydrocortisone sodium succinate 100mg powder and solvent for solution for injection vials |
| **34166** | Efcortelan soluble 50mg/ml Injection (Glaxo Laboratories Ltd) |
| **3754** | Hydrocortisone na succinate 100mg/vial Injection |
| **13350** | Hydrocortisone na succinate 500mg/vial Injection |
| **3651** | Solu-cortef 100mg/vial Injection (Pharmacia Ltd) |
| **17334** | Hydrocortisone 1% / Clioquinol 3% ointment |
| **1791** | Vioform-Hydrocortisone ointment (Novartis Consumer Health UK Ltd) |
| **11053** | Eurax Hc cream (Novartis Consumer Health UK Ltd) |
| **2696** | Eurax Hydrocortisone cream (Novartis Consumer Health UK Ltd) |
| **4881** | Hydrocortisone 0.25% / Crotamiton 10% cream |
| **49360** | Otosporin ear drops (Mawdsley-Brooks & Company Ltd) |
| **53721** | Otosporin ear drops (DE Pharmaceuticals) |
| **1247** | Otosporin ear drops (GlaxoSmithKline UK Ltd) |
| **50043** | Otosporin ear drops (Lexon (UK) Ltd) |
| **50499** | Generic Otosporin ear drops |
| **51421** | Otosporin ear drops (Stephar (U.K.) Ltd) |
| **1429** | Nystaform HC cream (Typharm Ltd) |
| **1457** | Terra-Cortril Nystatin cream (Pfizer Ltd) |
| **59806** | Pigmanorm cream (Imported (Germany)) |
| **22913** | Hydrocortisone with urea and lactic acid Cream |
| **6841** | Urea with hydrocortisone and lactic acid Cream |
| **2116** | Calmurid HC cream (Galderma (UK) Ltd) |
| **4687** | Methylprednisolone acetate with lidocaine 40mg/ml + 10mg/ml Injection |
| **925** | Depo-medrone with lidocaine 40mg/ml+10mg/ml Injection (Pharmacia Ltd) |
| **11134** | Betamethasone valerate 0.05% rectal ointment |
| **18069** | Germoloids HC spray (Bayer Plc) |
| **4238** | Perinal spray (Dermal Laboratories Ltd) |
| **10174** | Lidocaine 1% / Hydrocortisone 0.2% spray |
| **212** | Xyloproct 5%/0.275% ointment (AstraZeneca UK Ltd) |
| **5271** | Lidocaine 5% / Hydrocortisone acetate 0.275% ointment |
| **38353** | Loteprednol 0.5% eye drops |
| **38215** | Lotemax 0.5% eye drops (Bausch & Lomb UK Ltd) |
| **34111** | Hydrocortisone 0.1% emulsion |
| **2004** | Hydrocortisone 1% lotion |
| **2745** | Efcortelan 1% Cream (Glaxo Laboratories Ltd) |
| **22860** | Methylprednisolone with neomycin Cream |
| **3725** | Neo-medrone Cream (Pharmacia Ltd) |
| **10683** | Medrone 2mg tablets (Pfizer Ltd) |
| **2130** | Methylprednisolone 4mg tablets |
| **10684** | Methylprednisolone 2mg tablets |
| **10552** | Methylprednisolone 16mg tablets |
| **18042** | Medrone 100mg tablets (Pfizer Ltd) |
| **14172** | Methylprednisolone 100mg tablets |
| **8261** | Medrone 16mg tablets (Pfizer Ltd) |
| **15555** | Medrone 4mg tablets (Pfizer Ltd) |
| **35040** | Depo-Medrone 120mg/3ml suspension for injection vials (Pfizer Ltd) |
| **35688** | Methylprednisolone acetate 120mg/3ml suspension for injection vials |
| **14982** | Depo-Medrone 40mg/1ml suspension for injection vials (Pfizer Ltd) |
| **1133** | Depo-medrone 40mg/ml Injection (Pharmacia Ltd) |
| **48800** | Depo-Medrone 40mg/1ml suspension for injection vials (DE Pharmaceuticals) |
| **48748** | Depo-Medrone 40mg/1ml suspension for injection vials (Lexon (UK) Ltd) |
| **5493** | Methylprednisolone acetate 40mg/ml Injection |
| **48746** | Depo-Medrone 40mg/1ml suspension for injection vials (Mawdsley-Brooks & Company Ltd) |
| **35349** | Methylprednisolone acetate 80mg/2ml suspension for injection vials |
| **33132** | Methylprednisolone acetate 40mg/1ml suspension for injection vials |
| **27413** | Depo-Medrone 80mg/2ml suspension for injection vials (Pfizer Ltd) |
| **20157** | Depo-Medrone with Lidocaine suspension for injection 2ml vials (Pfizer Ltd) |
| **49076** | Depo-Medrone with Lidocaine suspension for injection 1ml vials (Lexon (UK) Ltd) |
| **7405** | Depo-Medrone with Lidocaine suspension for injection 1ml vials (Pfizer Ltd) |
| **35156** | Methylprednisolone 40mg/1ml / Lidocaine 10mg/1ml (1%) suspension for injection vials |
| **50253** | Depo-Medrone with Lidocaine suspension for injection 1ml vials (DE Pharmaceuticals) |
| **50734** | Depo-Medrone with Lidocaine suspension for injection 1ml vials (Mawdsley-Brooks & Company Ltd) |
| **35154** | Methylprednisolone 80mg/2ml / Lidocaine 20mg/2ml (1%) suspension for injection vials |
| **13397** | Methylprednisolone sodium succinate 1g powder and solvent for solution for injection vials |
| **25226** | Solu-Medrone 125mg powder and solvent for solution for injection vials (Pfizer Ltd) |
| **14188** | Methylprednisolone sodium succinate 500mg powder and solvent for solution for injection vials |
| **12405** | Methylprednisolone sodium succinate 2g powder and solvent for solution for injection vials |
| **18266** | Methylprednisolone sodium succinate 125mg powder and solvent for solution for injection vials |
| **18765** | Methylprednisolone sodium succinate 40mg powder and solvent for solution for injection vials |
| **25839** | Solu-Medrone 1g powder and solvent for solution for injection vials (Pfizer Ltd) |
| **23511** | Solu-Medrone 40mg powder and solvent for solution for injection vials (Pfizer Ltd) |
| **21540** | Solu-Medrone 500mg powder and solvent for solution for injection vials (Pfizer Ltd) |
| **5321** | Miconazole with hydrocortisone 2% with 1% Ointment |
| **37789** | Miconazole with hydrocortisone 2% with 1% Cream |
| **1677** | Miconazole with hydrocortisone 2% with 1% Cream |
| **11724** | Acorvio Plus cream (Ferndale Pharmaceuticals Ltd) |
| **11703** | Miconazole 2% / Fluprednidene 0.1% cream |
| **2625** | Hydrocortisone 1% / Miconazole 2% ointment |
| **9314** | Hydrocortisone 1% / Miconazole 2% cream |
| **6048** | Daktacort Hydrocortisone cream (McNeil Products Ltd) |
| **50759** | Daktacort 2%/1% cream (Sigma Pharmaceuticals Plc) |
| **929** | Daktacort 2%/1% cream (Janssen-Cilag Ltd) |
| **1430** | Daktacort ointment (Janssen-Cilag Ltd) |
| **51249** | Elocon 0.1% ointment (Mawdsley-Brooks & Company Ltd) |
| **2639** | Mometasone 0.1% scalp lotion |
| **60197** | Mometasone 0.1% ointment (Teva UK Ltd) |
| **53502** | Elocon 0.1% scalp lotion (Mawdsley-Brooks & Company Ltd) |
| **1838** | Elocon 0.1% cream (Merck Sharp & Dohme Ltd) |
| **50183** | Elocon 0.1% ointment (Lexon (UK) Ltd) |
| **1915** | Mometasone 0.1% cream |
| **60859** | Mometasone 0.1% ointment (Auden McKenzie (Pharma Division) Ltd) |
| **2638** | Elocon 0.1% scalp lotion (Merck Sharp & Dohme Ltd) |
| **49125** | Elocon 0.1% ointment (DE Pharmaceuticals) |
| **62153** | Mometasone 0.1% cream (Teva UK Ltd) |
| **1850** | Mometasone 0.1% ointment |
| **1224** | Elocon 0.1% ointment (Merck Sharp & Dohme Ltd) |
| **60287** | Mometasone 50micrograms/dose nasal spray (Sandoz Ltd) |
| **1095** | Nasonex 50micrograms/dose nasal spray (Merck Sharp & Dohme Ltd) |
| **1855** | Mometasone 50micrograms/dose nasal spray |
| **57517** | Nasonex 50micrograms/dose nasal spray (Waymade Healthcare Plc) |
| **51067** | Nasonex 50micrograms/dose nasal spray (Sigma Pharmaceuticals Plc) |
| **60266** | Mometasone 50micrograms/dose nasal spray (A A H Pharmaceuticals Ltd) |
| **53473** | Nasonex 50micrograms/dose nasal spray (Mawdsley-Brooks & Company Ltd) |
| **10254** | Mometasone 400micrograms/dose dry powder inhaler |
| **17590** | Asmanex 400micrograms/dose Twisthaler (Merck Sharp & Dohme Ltd) |
| **16018** | Mometasone 200micrograms/dose dry powder inhaler |
| **16433** | Asmanex 200micrograms/dose Twisthaler (Merck Sharp & Dohme Ltd) |
| **8459** | Silderm Cream (Wyeth Pharmaceuticals) |
| **4604** | Stiedex LPN 0.05% Cream (Stiefel Laboratories (UK) Ltd) |
| **14687** | Neomycin 0.5% / Betamethasone 0.1% eye ointment |
| **2438** | Betnesol-N eye ointment (Focus Pharmaceuticals Ltd) |
| **6883** | Betamethasone valerate 0.1% / Neomycin 0.5% ointment |
| **1553** | Betnovate-N ointment (Chemidex Pharma Ltd) |
| **4380** | Synalar N Ointment (GP Pharma) |
| **4869** | Synalar N cream (GP Pharma) |
| **10977** | Fluocinolone acetonide 0.025% / Neomycin 0.5% cream |
| **20884** | Fluocinolone acetonide 0.025% / Neomycin 0.5% ointment |
| **41452** | Synalar N ointment (Derma UK Ltd) |
| **42096** | Synalar N cream (Derma UK Ltd) |
| **18203** | Predsol -N Ear/eye drops solution (UCB Pharma Ltd) |
| **43088** | Predsol-N ear/eye drops (Focus Pharmaceuticals Ltd) |
| **18602** | Prednisolone 0.5% / Neomycin 0.5% ear/eye drops |
| **10403** | Adcortyl with graneodin Ointment (E R Squibb and Sons Ltd) |
| **1608** | Adcortyl with graneodin Cream (E R Squibb and Sons Ltd) |
| **17403** | Triamcinolone acetonide with neomycin nystatin and gramicidin Ointment |
| **23355** | Triamcinolone acetonide with gramicidin and neomycin Cream |
| **34165** | Betnovate -n Cream (Glaxo Laboratories Ltd) |
| **4854** | Triamcinolone acetonide with neomycin nystatin and gramicidin Cream |
| **38411** | Nystatin with hydrocortisone and neomycin Ointment |
| **10163** | Hydrocortisone with neomycin 1%+0.5% Ointment |
| **2763** | Hydrocortisone with neomycin 1%+0.5% Cream |
| **8353** | Neomycin sulphate with hydrocortisone Eye ointment |
| **30228** | Neo-cortef Ear/eye drops solution (Dominion Pharma) |
| **12723** | Neo-cortef Eye ointment (Dominion Pharma) |
| **15282** | Neo-cortef Eye ointment (PLIVA Pharma Ltd) |
| **15324** | Neo-cortef Ear drops (PLIVA Pharma Ltd) |
| **34147** | Neo-cortef Ear/eye drops solution (Dominion Pharma) |
| **2359** | Neo-cortef Ear drops (Dominion Pharma) |
| **15941** | Neo-cortef Ear drops (PLIVA Pharma Ltd) |
| **12653** | Neomycin sulphate with hydrocortisone Eye ointment |
| **12667** | Neomycin sulphate with hydrocortisone 0.5% + 1.5% Ear drops |
| **20583** | Hydrocortisone with neomycin 1%+0.5% Eye drops |
| **11964** | Neo-cortef Ear drops (Dominion Pharma) |
| **12769** | Hydrocortisone acetate with neomycin sulphate 1.5% with 0.5% ear drops |
| **12408** | Cloburate -n Eye drops (Dominion Pharma) |
| **17743** | Clobetasone butyrate with neomycin eye drops |
| **34628** | Betamethasone with neomycin Eye drops (C P Pharmaceuticals Ltd) |
| **4932** | Betnesol -n Nasal drops (UCB Pharma Ltd) |
| **11434** | Neomycin sulphate with betamethasone sodium phosphate 0.5% + 0.1% Eye drops |
| **11782** | Vista-methasone -n Ear drops (Martindale Pharmaceuticals Ltd) |
| **14894** | Vista-methasone -n Nasal drops (Martindale Pharmaceuticals Ltd) |
| **6340** | Betamethasone sodium phosphate 0.1% with neomycin 0.5% ear drops |
| **9735** | Betamethasone sodium phosphate 0.1% with neomycin 0.5% nasal drops |
| **21591** | Neomycin sulphate with betamethasone sodium phosphate 0.5% + 0.1% Eye drops |
| **4164** | Betamethasone sodium phosphate 0.1% with neomycin 0.5% eye drops |
| **25785** | Neomycin sulphate with betamethasone sodium phosphate 0.5% + 0.1% Eye/ear/nose drops |
| **6144** | Betnesol -n Ear drops (UCB Pharma Ltd) |
| **1226** | Betnesol -n Eye drops (UCB Pharma Ltd) |
| **1743** | Vista-methasone -n Eye drops (Martindale Pharmaceuticals Ltd) |
| **12961** | Neomycin 0.35% / Triamcinolone 0.1% ear drops |
| **4728** | Audicort ear drops (Mercury Pharma Group Ltd) |
| **9116** | Triamcinolone acetonide with nystatin Cream |
| **2852** | Nystadermal Cream (E R Squibb and Sons Ltd) |
| **1881** | Trimovate Ointment (Glaxo Laboratories Ltd) |
| **4656** | Clobetasone butyrate with oxytetracycline and nystatin cream |
| **1582** | Nystaform HC ointment (Typharm Ltd) |
| **54952** | Generic Timodine cream |
| **626** | Timodine cream (Alliance Pharmaceuticals Ltd) |
| **1203** | Tri-Adcortyl ointment (Bristol-Myers Squibb Pharmaceuticals Ltd) |
| **10391** | Terra-cortril Spray (Pfizer Ltd) |
| **22341** | Hydrocortisone with oxytetracycline Ointment |
| **12904** | Oxytetracycline with hydrocortisone & nystatin cream |
| **30604** | Hydrocortisone with nystatin and oxytetracycline Cream |
| **10972** | Oxytetracycline with hydrocortisone spray |
| **50941** | Terra-Cortril ointment (Intrapharm Laboratories Ltd) |
| **2266** | Oxytetracycline 3% / Hydrocortisone 1% ointment |
| **1326** | Terra-Cortril ointment (Pfizer Ltd) |
| **12515** | Gregoderm ointment (Unigreg Ltd) |
| **15590** | Hydrocortisone with neomycin nystatin and polymyxin Ointment |
| **14051** | Dexamethasone with neomycin with polymyxin eye ointment |
| **5006** | Dexamethasone with neomycin with polymyxin eye drops |
| **8618** | Quinocort Cream (Quinoderm Ltd) |
| **54178** | Quinocort cream (Ecolab Healthcare Division) |
| **8497** | Hydrocortisone 1% / Potassium hydroxyquinoline sulfate 0.5% cream |
| **25272** | Precortisyl 1mg Tablet (Hoechst Marion Roussel) |
| **34631** | Prednisolone 1mg Tablet (Co-Pharma Ltd) |
| **34914** | Prednisolone 1mg Tablet (Celltech Pharma Europe Ltd) |
| **2368** | Prednisolone 2.5mg tablet |
| **28376** | Prednisolone 2.5mg Gastro-resistant tablet (Biorex Laboratories Ltd) |
| **34109** | Prednisolone 5 mg gastro-resistant tablet |
| **45302** | Prednisolone 5mg Tablet (Biorex Laboratories Ltd) |
| **28859** | Deltastab 5mg Tablet (Waymade Healthcare Plc) |
| **33691** | Prednisolone 5mg Gastro-resistant tablet (Biorex Laboratories Ltd) |
| **38407** | Prednisolone 20mg tablet |
| **23512** | Precortisyl 5mg Tablet (Hoechst Marion Roussel) |
| **27962** | Deltastab 1mg Tablet (Waymade Healthcare Plc) |
| **20095** | Precortisyl forte 25mg Tablet (Aventis Pharma) |
| **33990** | Prednisolone 5mg Tablet (IVAX Pharmaceuticals UK Ltd) |
| **33988** | Prednisolone 5mg Tablet (Co-Pharma Ltd) |
| **2704** | Prednisolone 25mg tablets |
| **53336** | Prednisolone 25mg tablets (A A H Pharmaceuticals Ltd) |
| **41745** | Prednisolone 25mg tablets (Zentiva) |
| **58234** | Prednisolone 10mg/5ml oral solution |
| **59338** | Prednisolone 1mg/5ml oral solution |
| **54434** | Prednisolone 2.5mg/5ml oral suspension |
| **54118** | Prednisolone 25mg/5ml oral suspension |
| **55024** | Prednisolone 5mg/5ml oral solution |
| **53313** | Prednisolone 20mg/5ml oral suspension |
| **41515** | Prednisolone 5mg tablets (Teva UK Ltd) |
| **60421** | Prednisolone 5mg tablets (Co-Pharma Ltd) |
| **32803** | Prednisolone 5mg gastro-resistant tablets (Actavis UK Ltd) |
| **34393** | Prednisolone 5mg gastro-resistant tablets (Teva UK Ltd) |
| **58987** | Prednisolone 5mg gastro-resistant tablets (Phoenix Healthcare Distribution Ltd) |
| **59229** | Dilacort 5mg gastro-resistant tablets (Auden McKenzie (Pharma Division) Ltd) |
| **34748** | Prednisolone 1mg tablets (Teva UK Ltd) |
| **28375** | Prednisolone 2.5mg gastro-resistant tablets (A A H Pharmaceuticals Ltd) |
| **34978** | Prednisolone 1mg tablets (Wockhardt UK Ltd) |
| **578** | Prednisolone 1mg tablets |
| **34781** | Prednisolone 5mg tablets (Kent Pharmaceuticals Ltd) |
| **34452** | Prednisolone 1mg tablets (A A H Pharmaceuticals Ltd) |
| **44** | Prednisolone 5mg gastro-resistant tablets |
| **5913** | Deltacortril 2.5mg gastro-resistant tablets (Alliance Pharmaceuticals Ltd) |
| **31532** | Prednisolone 5mg gastro-resistant tablets (A A H Pharmaceuticals Ltd) |
| **61162** | Prednisolone 5mg tablets (Waymade Healthcare Plc) |
| **21417** | Prednisolone 5mg tablets (A A H Pharmaceuticals Ltd) |
| **34404** | Prednisolone 1mg tablets (Actavis UK Ltd) |
| **55480** | Prednisolone 2.5mg gastro-resistant tablets (Alliance Pharmaceuticals Ltd) |
| **5490** | Deltacortril 5mg gastro-resistant tablets (Alliance Pharmaceuticals Ltd) |
| **51753** | Prednisolone 1mg tablets (Co-Pharma Ltd) |
| **95** | Prednisolone 5mg tablets |
| **56891** | Prednisolone 1mg tablets (Waymade Healthcare Plc) |
| **61132** | Prednisolone 1mg tablets (Boston Healthcare Ltd) |
| **32835** | Prednisolone 5mg tablets (Wockhardt UK Ltd) |
| **34461** | Prednisolone 2.5mg gastro-resistant tablets (Actavis UK Ltd) |
| **59912** | Prednisolone 5mg gastro-resistant tablets (Waymade Healthcare Plc) |
| **557** | Prednisolone 2.5mg gastro-resistant tablets |
| **58000** | Prednisolone 5mg tablets (Almus Pharmaceuticals Ltd) |
| **58369** | Prednisolone 5mg tablets (Boston Healthcare Ltd) |
| **29333** | Prednisolone 5mg tablets (Actavis UK Ltd) |
| **34660** | Prednisolone 1mg tablets (Kent Pharmaceuticals Ltd) |
| **59283** | Dilacort 2.5mg gastro-resistant tablets (Auden McKenzie (Pharma Division) Ltd) |
| **58384** | Prednisolone 1mg tablets (Almus Pharmaceuticals Ltd) |
| **34221** | Prednisolone suppositories |
| **42408** | Prednisolone 40mg/100ml enema |
| **820** | Prednisolone 20mg/100ml retention enema |
| **10934** | Predenema 20mg/100ml long tube (Forest Laboratories UK Ltd) |
| **61052** | Prednisolone 20mg/100ml enema long tube |
| **9727** | Prednisolone 50mg tablets |
| **53370** | Pred Forte 1% eye drops (DE Pharmaceuticals) |
| **1897** | Pred Forte 1% eye drops (Allergan Ltd) |
| **2781** | Prednisolone acetate 1% eye drops |
| **2596** | Prednisolone acetate 0.5% eye drops |
| **50888** | Pred Forte 1% eye drops (Necessity Supplies Ltd) |
| **8306** | Prednisolone 25mg/1ml suspension for injection ampoules |
| **18660** | Deltastab 25mg/1ml suspension for injection ampoules (AMCo) |
| **15164** | Cinchocaine 1mg / Prednisolone hexanoate 1.3mg suppositories |
| **2857** | Scheriproct suppositories (Bayer Plc) |
| **6805** | Prednisolone 20mg/application foam enema |
| **1907** | Predfoam 20mg/application enema (Chemidex Pharma Ltd) |
| **59742** | Prednisolone sodium phosphate 0.1% ear drops |
| **14972** | Prednisolone sodium phosphate 0.5% ear/eye drops |
| **61916** | Prednisolone sodium phosphate 0.3% ear drops |
| **42947** | Predsol 0.5% ear/eye drops (Focus Pharmaceuticals Ltd) |
| **31830** | Prednisolone 0.05% Eye drops (Huddersfield Royal Infirmary) |
| **11176** | Prednisolone sodium phosphate 0.5% eye drops |
| **14327** | Predsol Ear/eye drops solution (UCB Pharma Ltd) |
| **5063** | Prednisolone sodium phosphate 0.5% eye drops |
| **1460** | Predsol Eye drops (UCB Pharma Ltd) |
| **11083** | Predsol Ear drops (UCB Pharma Ltd) |
| **38117** | Prednisolone sodium phosphate eye drops |
| **3544** | Minims prednisolone sodium phosphate 0.5% eye drops 0.5ml unit dose (Bausch & Lomb UK Ltd) |
| **37271** | Prednisolone sodium phosphate 0.01% eye drops |
| **5632** | Prednisolone sodium phosphate 0.5% eye drops 0.5ml unit dose preservative free |
| **57777** | Prednisolone sodium phosphate 0.01% eye drops preservative free |
| **6701** | Prednisolone sodium phosphate 0.1% eye drops |
| **56270** | Prednisolone sodium phosphate 0.1% eye drops preservative free (Drug Tariff Special Order) |
| **44697** | Prednisolone sodium phosphate 1% eye drops preservative free |
| **37246** | Prednisolone sodium phosphate 0.03% eye drops |
| **6791** | Prednisolone sodium phosphate 0.3% eye drops |
| **37499** | Prednisolone sodium phosphate 0.125% eye drops |
| **19960** | Prednisolone sodium phosphate 0.3% eye drops preservative free |
| **38063** | Prednisolone sodium phosphate preservative free eye drops |
| **35324** | Prednisolone sodium phosphate 0.03% eye drops |
| **53007** | Prednisolone sodium phosphate 0.125% eye drops preservative free |
| **20996** | Prednisolone sodium phosphate 0.1% eye drops preservative free |
| **10028** | Prednisolone sodium phosphate 0.05% eye drops |
| **24224** | Codelsol 16mg/ml Injection (MSD Thomas Morson Pharmaceuticals) |
| **47142** | Prednisolone 5mg Soluble tablet (Amdipharm Plc) |
| **1063** | Prednesol 5mg Tablet (Sovereign Medical Ltd) |
| **61689** | Prednisolone 5mg soluble tablets (A A H Pharmaceuticals Ltd) |
| **19141** | Prednisolone 5mg soluble tablets (AMCo) |
| **955** | Prednisolone 5mg soluble tablets |
| **2342** | Predsol 20mg/100ml retention enema (Focus Pharmaceuticals Ltd) |
| **48320** | Prednisolone 20mg/100ml enema standard tube |
| **2881** | Predsol 5mg suppositories (Focus Pharmaceuticals Ltd) |
| **3913** | Prednisolone sodium phosphate 5mg suppositories |
| **15175** | Predenema 20mg/100ml standard tube (Forest Laboratories UK Ltd) |
| **31327** | Prednisolone steaglate 6.65mg tablet |
| **3345** | Sintisone Tablet (Pharmacia Ltd) |
| **44802** | Lodotra 5mg modified-release tablets (Napp Pharmaceuticals Ltd) |
| **44723** | Prednisone 5mg modified-release tablets |
| **44380** | Prednisone 1mg modified-release tablets |
| **54432** | Lodotra 1mg modified-release tablets (Napp Pharmaceuticals Ltd) |
| **58061** | Prednisone 50mg tablets |
| **44803** | Lodotra 2mg modified-release tablets (Napp Pharmaceuticals Ltd) |
| **3557** | Prednisone 1mg tablets |
| **46711** | Prednisone 2mg modified-release tablets |
| **2949** | Prednisone 5mg tablets |
| **43544** | Prednisone 5mg Tablet (Knoll Ltd) |
| **21833** | Decortisyl 5mg Tablet (Roussel Laboratories Ltd) |
| **45314** | Salicylic acid 5% with propylene glycol in clobetasol propionate 47.5% cream |
| **5110** | Rimexolone 10mg/ml eye drops |
| **15213** | Vexol 10mg/ml eye drops (Alcon Laboratories (UK) Ltd) |
| **1801** | Ventide inhaler (GlaxoSmithKline UK Ltd) |
| **11307** | Salbutamol 100micrograms/dose / Beclometasone 50micrograms/dose inhaler |
| **5096** | Betamethasone dipropionate 0.05% / Salicylic acid 2% scalp application |
| **1717** | Diprosalic 0.05%/2% scalp application (Merck Sharp & Dohme Ltd) |
| **54741** | Diprosalic 0.05%/2% scalp application (Lexon (UK) Ltd) |
| **52476** | Diprosalic 0.05%/2% scalp application (Waymade Healthcare Plc) |
| **10571** | Stiedex lotion (Stiefel Laboratories (UK) Ltd) |
| **21267** | Salicylic acid 1% / Desoximetasone 0.25% lotion |
| **6616** | Salmeterol 25micrograms with fluticasone 50micrograms CFC free inhaler |
| **5942** | Salmeterol 50micrograms with fluticasone 250micrograms CFC free inhaler |
| **5864** | Salmeterol 25micrograms with fluticasone 250micrograms CFC free inhaler |
| **6938** | Salmeterol 50micrograms with fluticasone 100micrograms dry powder inhaler |
| **5558** | Salmeterol 50micrograms with fluticasone 500micrograms CFC free inhaler |
| **6569** | Salmeterol 25micrograms with fluticasone 125micrograms CFC free inhaler |
| **51394** | Seretide 500 Accuhaler (Waymade Healthcare Plc) |
| **13273** | Fluticasone propionate 100micrograms/dose / Salmeterol 50micrograms/dose dry powder inhaler |
| **51861** | Seretide 500 Accuhaler (Mawdsley-Brooks & Company Ltd) |
| **11410** | Fluticasone propionate 500micrograms/dose / Salmeterol 50micrograms/dose dry powder inhaler |
| **62126** | Seretide 100 Accuhaler (DE Pharmaceuticals) |
| **3666** | Seretide 500 Accuhaler (GlaxoSmithKline UK Ltd) |
| **55677** | Seretide 500 Accuhaler (Lexon (UK) Ltd) |
| **665** | Seretide 100 Accuhaler (GlaxoSmithKline UK Ltd) |
| **51593** | Seretide 500 Accuhaler (DE Pharmaceuticals) |
| **53283** | Seretide 100 Accuhaler (Waymade Healthcare Plc) |
| **8326** | Actinac lotion (Peckforton Pharmaceuticals Ltd) |
| **11628** | Dexamethasone 0.1% / Tobramycin 0.3% eye drops |
| **6463** | Tobradex 3mg/ml / 1mg/ml eye drops (Alcon Laboratories (UK) Ltd) |
| **23928** | Tramazoline with dexamethasone 120 micrograms + 20 micrograms Nasal spray suspension |
| **5273** | Dexa-Rhinaspray Duo nasal spray (Boehringer Ingelheim Ltd) |
| **6905** | Tramazoline 120micrograms/dose / Dexamethasone 20micrograms/dose nasal spray |
| **46738** | Pigmanorm Cream (Louis Widmer) |
| **46855** | Tretinoin with hydrocortisone and hydroquinone 0.1% + 1% + 5% cream |
| **18300** | Ledercort Cream (Wyeth Pharmaceuticals) |
| **3862** | Adcortyl 0.10% Cream (E R Squibb and Sons Ltd) |
| **10732** | Triamcinolone acetonide Ointment |
| **3510** | Adcortyl 0.10% Ointment (E R Squibb and Sons Ltd) |
| **4444** | Triamcinolone acetonide Cream |
| **12074** | Ledercort Ointment (Wyeth Pharmaceuticals) |
| **1085** | Nasacort 55micrograms/dose nasal spray (Sanofi) |
| **1799** | Triamcinolone 55micrograms/dose nasal spray |
| **40446** | Nasacort Allergy 55micrograms/dose nasal spray (Sanofi) |
| **13981** | Adcortyl 10mg/ml Intradermal injection (E R Squibb and Sons Ltd) |
| **9368** | Triamcinolone acetonide 10mg/ml IA/ID |
| **35578** | Triamcinolone acetonide 50mg/5ml suspension for injection vials |
| **14958** | Triamcinolone acetonide 10mg/1ml suspension for injection ampoules |
| **14962** | Adcortyl Intra-articular / Intradermal 50mg/5ml suspension for injection vials (Bristol-Myers Squibb Pharmaceuticals Ltd) |
| **14335** | Adcortyl Intra-articular / Intradermal 10mg/1ml suspension for injection ampoules (Bristol-Myers Squibb Pharmaceuticals Ltd) |
| **11123** | Triamcinolone acetonide 10mg/ml IA/ID |
| **8864** | Adcortyl 10mg/ml Intraarticular / intradermal injection (E R Squibb and Sons Ltd) |
| **4488** | Triamcinolone acetonide 40mg/ml IA/IM |
| **16582** | Triamcinolone acetonide 40mg/ml suspension for injection |
| **768** | Kenalog 40mg/ml Injection (E R Squibb and Sons Ltd) |
| **48406** | Triamcinolone acetonide 40mg/1ml suspension for injection vials |
| **4123** | Kenalog 40mg/ml Intraarticular injection (E R Squibb and Sons Ltd) |
| **37737** | Kenalog 40mg/ml Injection (E R Squibb and Sons Ltd) |
| **16583** | Kenalog Intra-articular / Intramuscular 40mg/1ml suspension for injection vials (Bristol-Myers Squibb Pharmaceuticals Ltd) |
| **30244** | Triamcinolone acetonide 40mg/ml injection |
| **3703** | Kenalog 80mg/2ml Intramuscular injection (E R Squibb and Sons Ltd) |
| **4125** | Triamcinolone acetonide 80mg/2ml intramuscular injection |
| **15617** | Ledercort 4mg Tablet (Wyeth Pharmaceuticals) |
| **24014** | Ledercort 2mg Tablet (Wyeth Pharmaceuticals) |
| **19908** | Triamcinolone 2mg Tablet |
| **23111** | Triamcinolone 4mg Tablet |
| **990** | Adcortyl in Orabase 0.1% oromucosal paste (Bristol-Myers Squibb Pharmaceuticals Ltd) |
| **10216** | Adcortyl In Orabase for Mouth Ulcer 0.1% oromucosal paste (Bristol-Myers Squibb Pharmaceuticals Ltd) |
| **50216** | Kenalog Intramuscular 40mg/1ml suspension for injection pre-filled syringes (Bristol-Myers Squibb Pharmaceuticals Ltd) |
| **22047** | Kenalog Intramuscular 80mg/2ml suspension for injection pre-filled syringes (Bristol-Myers Squibb Pharmaceuticals Ltd) |
| **1917** | Triamcinolone 0.1% oromucosal paste |
| **33131** | Triamcinolone acetonide 80mg/2ml suspension for injection pre-filled syringes |
| **50026** | Triamcinolone acetonide 40mg/1ml suspension for injection pre-filled syringes |
| **1607** | Aureocort Ointment (Wyeth Pharmaceuticals) |
| **6342** | Aureocort ointment (AMCo) |
| **7187** | Triamcinolone acetonide 0.1% / Chlortetracycline 3.09% ointment |
| **1771** | Tri-Adcortyl Otic ointment (Bristol-Myers Squibb Pharmaceuticals Ltd) |
| **1205** | Tri-Adcortyl cream (Bristol-Myers Squibb Pharmaceuticals Ltd) |
| **7992** | Lederspan 5mg/ml Injection (Wyeth Pharmaceuticals) |
| **15016** | Triamcinolone hexacetonide 5mg/ml Injection |
| **50854** | Lederspan 100mg/5ml suspension for injection vials (Wyeth Pharmaceuticals) |
| **50853** | Lederspan 20mg/1ml suspension for injection vials (Wyeth Pharmaceuticals) |
| **57856** | Triamcinolone hexacetonide 20mg/1ml suspension for injection vials |
| **10479** | Sential HC Cream (Galderma (UK) Ltd) |
| **1880** | Alphaderm 1%/10% cream (Alliance Pharmaceuticals Ltd) |
| **45725** | Hydromol HC Intensive cream (Alliance Pharmaceuticals Ltd) |
| **10220** | Hydrocortisone 1% / Urea 10% cream |
| **18222** | Coal tar with hydrocortisone ointment |
| **19179** | Benzyl benzoate with hydrocortisone and soothing agents suppository |
| **11889** | Hydrocortisone with benzyl benzoate and soothing agents Ointment |
| **11293** | Hydrocortisone with benzyl benzoate and soothing agents Cream |
| **6697** | Hydrocortisone with benzyl benzoate and soothing agents Suppository |
| **18005** | Hydrocortisone with benzyl benzoate and soothing agents Suppository |
| **59611** | Hydrocortisone 2.5% / Tretinoin 0.1% / Hydroquinone 5% cream |
| **56295** | Beclometasone 0.025% in White soft paraffin |
| **49409** | Beclometasone 0.0025% in White soft paraffin |
| **51936** | Prednisolone sodium phosphate 0.03% eye drops preservative free |
| **53731** | Dexamethasone 1% eye drops preservative free |
| **50436** | Prednisolone sodium phosphate 0.5% eye drops preservative free |
| **58784** | Dexamethasone 0.1% eye drops 0.4ml unit dose preservative free |
| **61954** | Dexafree 1mg/1ml eye drops 0.4ml unit dose (Spectrum Thea Pharmaceuticals Ltd) |
| **61958** | Dexamethasone 3.8mg/1ml solution for injection vials |
| **56940** | Dexamethasone 6.6mg/2ml solution for injection ampoules |
| **58592** | Plenadren 20mg modified-release tablets (ViroPharma Ltd) |
| **54794** | Hydrocortisone 20mg modified-release tablets |
| **51871** | Hydrocortisone 2mg capsules |
| **53953** | Hydrocortisone 5mg modified-release tablets |
| **59418** | Plenadren 5mg modified-release tablets (ViroPharma Ltd) |
| **52053** | Hydrocortisone 3mg/5ml oral suspension |
| **51849** | Hydrocortisone 1mg/5ml oral suspension |
| **49412** | Clenil Modulite 200micrograms/dose inhaler (Mawdsley-Brooks & Company Ltd) |
| **61644** | Fostair NEXThaler 100micrograms/dose / 6micrograms/dose dry powder inhaler (Chiesi Ltd) |
| **62030** | Beclometasone 100micrograms/dose / Formoterol 6micrograms/dose dry powder inhaler |
| **51209** | Fluticasone 125micrograms/dose / Formoterol 5micrograms/dose inhaler CFC free |
| **48666** | Flutiform 250micrograms/dose / 10micrograms/dose inhaler (Napp Pharmaceuticals Ltd) |
| **50036** | Flutiform 125micrograms/dose / 5micrograms/dose inhaler (Napp Pharmaceuticals Ltd) |
| **49868** | Fluticasone 250micrograms/dose / Formoterol 10micrograms/dose inhaler CFC free |
| **56443** | Dexamethasone 10mg/5ml oral solution |
| **60064** | Dexamethasone 10mg/5ml oral solution sugar free |
| **14412** | DESONIDE .05 % CRE |
| **22074** | FLUNISOLIDE NASAL |

**Supplementary Table S4:** Read and ICD10 codes for infectious mononucleosis

| **Read Code** | **Term** |
| --- | --- |
| **A79y.11** | Epstein-Barr virus |
| **C396.00** | Immunodef follow hereditary defect respon Epstein-Barr vir |
| **A750.00** | Gammaherpesviral mononucleosis |
| **J631200** | Hepatitis in infectious mononucleosis |
| **A751.00** | Cytomegaloviral mononucleosis |
| **AyuD500** | [X]Infectious mononucleosis, unspecified |
| **A75..00** | Infectious mononucleosis |
| **AyuD400** | [X]Other infectious mononucleosis |
| **A75..11** | Glandular fever |
| **A752.00** | Pfeiffer's disease |
| **A75..12** | Pfeiffer's disease |
| **43A2.00** | Inf mononucleos test positive |
| **43A2.11** | Monospot test positive |
| **43F4100** | Heterophile agglutin test abnormal |
| **43jV.00** | Epstein-Barr virus nucleic acid detection |
| **ICD10 Code** | **Term** |
| **B27** | Infectious mononucleosis |
| **B27.0** | Gammaherpesviral mononucleosis |
| **B27.1** | Cytomegaloviral mononucleosis |
| **B27.8** | Other infectious mononucleosis |
| **B27.9** | Infectious mononucleosis, unspecified |
| **D82.3** | Immunodeficiency following hereditary defective response to Epstein-Barr virus |

**Supplementary Table S5:** Read and ICD10 codes for immunosuppression

| **Read Code** | **Term** |
| --- | --- |
| **43C3.00** | HTLV-3 antibody positive |
| **43C3.11** | HIV positive |
| **65QA.00** | AIDS carrier |
| **65VE.00** | Notification of AIDS |
| **66j..00** | Human immunodeficiency virus monitoring |
| **66j0.00** | Human immunodeficiency virus annual review |
| **A788.00** | Acquired immune deficiency syndrome |
| **A788.11** | Human immunodeficiency virus infection |
| **A788000** | Acute human immunodeficiency virus infection |
| **A788100** | Asymptomatic human immunodeficiency virus infection |
| **A788200** | HIV infection with persistent generalised lymphadenopathy |
| **A788300** | Human immunodeficiency virus with constitutional disease |
| **A788400** | Human immunodeficiency virus with neurological disease |
| **A788500** | Human immunodeficiency virus with secondary infection |
| **A788600** | Human immunodeficiency virus with secondary cancers |
| **A788U00** | HIV disease result/haematological+immunologic abnorms,NEC |
| **A788W00** | HIV disease resulting in unspecified malignant neoplasm |
| **A788X00** | HIV disease resulting/unspcf infectious+parasitic disease |
| **A788y00** | Human immunodeficiency virus with other clinical findings |
| **A788z00** | Acquired human immunodeficiency virus infection syndrome NOS |
| **A789.00** | Human immunodef virus resulting in other disease |
| **A789000** | HIV disease resulting in mycobacterial infection |
| **A789100** | HIV disease resulting in cytomegaloviral disease |
| **A789200** | HIV disease resulting in candidiasis |
| **A789300** | HIV disease resulting in Pneumocystis carinii pneumonia |
| **A789311** | HIV disease resulting in Pneumocystis jirovecii pneumonia |
| **A789400** | HIV disease resulting in multiple infections |
| **A789500** | HIV disease resulting in Kaposi's sarcoma |
| **A789511** | HIV disease resulting in Kaposi sarcoma |
| **A789600** | HIV disease resulting in Burkitt's lymphoma |
| **A789700** | HIV dis resulting oth types of non-Hodgkin's lymphoma |
| **A789800** | HIV disease resulting in multiple malignant neoplasms |
| **A789900** | HIV disease resulting in lymphoid interstitial pneumonitis |
| **A789A00** | HIV disease resulting in wasting syndrome |
| **A789X00** | HIV dis reslt/oth mal neopl/lymph,h'matopoetc+reltd tissu |
| **AyuC.00** | [X]Human immunodeficiency virus disease |
| **AyuC100** | [X]HIV disease resulting in other viral infections |
| **AyuC300** | [X]HIV disease resulting in multiple infections |
| **AyuC400** | [X]HIV disease resulting/other infectious+parasitic diseases |
| **AyuC600** | [X]HIV disease resulting in other non-Hodgkin's lymphoma |
| **AyuCB00** | [X]HIV disease result/haematological+immunologic abnorms,NEC |
| **AyuCC00** | [X]HIV disease resulting in other specified conditions |
| **AyuCD00** | [X]Unspecified human immunodeficiency virus [HIV] disease |
| **C30yy11** | Adenosine-deaminase deficiency |
| **C390.00** | Deficiencies of humoral immunity |
| **C390.11** | Agammaglobulinaemia |
| **C390000** | Hypogammaglobulinaemia NOS |
| **C390100** | Selective IgA immunodeficiency |
| **C390200** | Selective IgM immunodeficiency |
| **C390300** | Selective IgG immunodeficiency |
| **C390400** | Other selective immunoglobulin deficiency |
| **C390500** | Congenital hypogammaglobulinaemia |
| **C390511** | Bruton's agammaglobulinaemia |
| **C390512** | Congenital X-linked agammaglobulinaemia |
| **C390600** | Immunodeficiency with IgM hypergammaglobulinaemia |
| **C390700** | Common variable immunodeficiency |
| **C390800** | Transient infant hypogammaglobulinaemia |
| **C390900** | Agammaglobulinaemia NEC |
| **C390A00** | Dysimmunoglobulinaemia NEC |
| **C390A11** | Dysgammaglobulinaemia NEC |
| **C390B00** | Antibod def wth nr-norm imunoglob/or wth hyperimunoglobaemia |
| **C390y00** | Other specified deficiency of humoral immunity |
| **C390z00** | Deficiency of humoral immunity NOS |
| **C391.00** | Deficiencies of cell-mediated immunity |
| **C391000** | Predominantly T-cell immuno-deficiency NOS |
| **C391011** | T-lymphocyte deficiency |
| **C391012** | Cellular immunity syndrome |
| **C391100** | Di George syndrome |
| **C391200** | Wiskott - Aldrich syndrome |
| **C391211** | Thrombocytopenic eczema with immunodeficiency |
| **C392.00** | Combined immunity deficiency |
| **C392100** | Severe combined immunodeficiency |
| **C392111** | Swiss type agammaglobulinaemia |
| **C392300** | Severe combined immunodefiency with reticular dysgenesis |
| **C392400** | Severe combined immunodef with low T- and B-cell numbers |
| **C392500** | Severe combined immunodef with low or normal B-cell numbers |
| **C392600** | Adenosine deaminase deficiency |
| **C392700** | Purine nucleoside phosphorylase deficiency |
| **C392800** | Major histocompatibility complex class I deficiency |
| **C392900** | Major histocompatibility complex class II deficiency |
| **C392z00** | Combined immunity deficiency NOS |
| **C393.00** | Unspecified immunity deficiency |
| **C395.00** | Immunodeficiency with short-limbed stature |
| **C396.00** | Immunodef follow hereditary defect respon Epstein-Barr vir |
| **C397.00** | Hyperimmunoglobulin E syndrome |
| **C398.00** | Common variable immunodeficiency |
| **C398000** | Com var immunodef with predom abn B-cell numbers and functns |
| **C398200** | Common variable immunodef wth autoantibod to B- or T-cells |
| **C399.00** | Defects in the complement system |
| **C39X.00** | Immunodeficiency associated+major defect, unspecified |
| **C39y000** | Lymphocyte function antigen-1 defect |
| **Cyu0000** | [X]Other immunodeficiencies+predominantly antibody defects |
| **Cyu0400** | [X]Other common variable immunodeficiencies |
| **Cyu0500** | [X]Other specified immunodeficiency disorders |
| **D41y200** | Pseudocholinesterase deficiency |
| **Eu02400** | [X]Dementia in human immunodef virus [HIV] disease |
| **L179.00** | HIV disease complicating pregnancy childbirth puerperium |
| **R109.00** | [D]Laboratory evidence of human immunodeficiency virus [HIV] |
| **7901000** | Allotransplantation of heart NEC |
| **7900.00** | Transplantation of heart and lung |
| **7B00.00** | Transplantation of kidney |
| **7800.00** | Transplantation of liver |
| **7901.00** | Other transplantation of heart |
| **7B00z00** | Transplantation of kidney NOS |
| **ZV42000** | [V]Kidney transplanted |
| **SP08600** | Liver transplant failure and rejection |
| **ZV42700** | [V]Liver transplanted |
| **ZV42100** | [V]Heart transplanted |
| **ZV42600** | [V]Lung transplanted |
| **7450.00** | Transplantation of lung |
| **SP08100** | Transplanted organ rejection |
| **SP08300** | Kidney transplant failure and rejection |
| **7B00100** | Transplantation of kidney from live donor |
| **TB00111** | Renal transplant with complication, without blame |
| **ZV42.00** | [V]Transplanted organ or tissue |
| **7B00200** | Transplantation of kidney from cadaver |
| **SP08z00** | Transplanted organ complication NOS |
| **7B06300** | Exploration of renal transplant |
| **7800z00** | Transplantation of liver NOS |
| **SP08500** | Heart-lung transplant failure and rejection |
| **SP08000** | Transplanted organ failure |
| **TB00200** | Liver transplant with complication, without blame |
| **7800000** | Orthotopic transplantation of liver |
| **7830.00** | Transplantation of pancreas |
| **14S8.00** | H/O: liver recipient |
| **7450z00** | Transplantation of lung NOS |
| **7901z00** | Other transplantation of heart NOS |
| **ZV42y12** | [V]Pancreas transplanted |
| **SP08.00** | Transplanted organ complication |
| **SP08400** | Heart transplant failure and rejection |
| **7B01500** | Transplant nephrectomy |
| **14S2.00** | H/O: kidney recipient |
| **7900000** | Allotransplantation of heart and lung |
| **TB00100** | Kidney transplant with complication, without blame |
| **7B00000** | Autotransplant of kidney |
| **7830100** | Transplantation of whole pancreas |
| **14S3.00** | H/O: heart recipient |
| **7830300** | Transplantation of islets of Langerhans |
| **7900z00** | Transplantation of heart and lung NOS |
| **TB00000** | Heart transplant with complication, without blame |
| **14S9.00** | H/O: lung recipient |
| **7B00111** | Allotransplantation of kidney from live donor |
| **7830z00** | Transplantation of pancreas NOS |
| **7800200** | Replacement of previous liver transplant |
| **7901y00** | Other specified other transplantation of heart |
| **SP08011** | Det.ren.func.after ren.transpl |
| **7B00y00** | Other specified transplantation of kidney |
| **7800100** | Heterotopic transplantation of liver |
| **7B01511** | Excision of rejected transplanted kidney |
| **7901100** | Xenotransplantation of heart |
| **7450y00** | Other specified transplantation of lung |
| **7800111** | Auxillary liver transplant |
| **7B00300** | Allotransplantation of kidney from cadaver, heart-beating |
| **7B0F100** | Pre-transplantation of kidney work-up, recipient |
| **7B0F.00** | Interventions associated with transplantation of kidney |
| **7450100** | Single lung transplant |
| **ZV42y11** | [V]Intestine transplanted |
| **7901500** | Revision of transplantation of heart NEC |
| **7B0F400** | Post-transplantation of kidney examination, live donor |
| **7B0F200** | Pre-transplantation of kidney work-up, live donor |
| **7831200** | Excision of transplanted pancreas |
| **7B00400** | Allotransplantation kidney from cadaver, heart non-beating |
| **ZV42.11** | [V]Transplanted organ |
| **7450000** | Double lung transplant |
| **7800500** | Orthotopic transplantation of liver NEC |
| **7B00211** | Allotransplantation of kidney from cadaver |
| **7800y00** | Other specified transplantation of liver |
| **7800112** | Piggy back liver transplant |
| **764C.00** | Transplantation of ileum |
| **7830200** | Transplantation of tail of pancreas |
| **7125.00** | Transplantation of thymus gland |
| **7B0F300** | Post-transplantation of kidney examination, recipient |
| **7B0Fz00** | Interventions associated with transplantation of kidney NOS |
| **7B0Fy00** | OS interventions associated with transplantation of kidney |
| **SP08H00** | Acute rejection of renal transplant |
| **SP08G00** | Acute rejection of renal transplant - grade III |
| **SP08D00** | Acute-on-chronic rejection of renal transplant |
| **SP08E00** | Acute rejection of renal transplant - grade I |
| **7B00212** | Cadaveric renal transplant |
| **7800400** | Orthotopic transplantation of whole liver |
| **SP08N00** | Unexplained episode of renal transplant dysfunction |
| **7B00600** | Xenograft renal transplant |
| **SP08R00** | Renal transplant rejection |
| **7842000** | Transplantation of spleen |
| **SP08P00** | Stenosis of vein of transplanted kidney |
| **SP08J00** | Chronic rejection of renal transplant |
| **SP08W00** | Vascular complication of renal transplant |
| **SP08F00** | Acute rejection of renal transplant - grade II |
| **7901300** | Piggyback transplantation of heart |
| **SP08T00** | Urological complication of renal transplant |
| **SP08900** | Complication of transplanted lung |
| **SP08V00** | Very mild acute rejection of renal transplant |
| **9b8B200** | Cardiothoracic transplantation |
| **7B00500** | Allotransplantation of kidney from cadaver NEC |
| **761N.00** | Transplantation of stomach |
| **ICD10 Code** | **Term** |
| **B20** | Human immunodeficiency virus [HIV] disease resulting in infectious and parasitic diseases |
| **B20.0** | HIV disease resulting in mycobacterial infection |
| **B20.1** | HIV disease resulting in other bacterial infections |
| **B20.2** | HIV disease resulting in cytomegaloviral disease |
| **B20.3** | HIV disease resulting in other viral infections |
| **B20.4** | HIV disease resulting in candidiasis |
| **B20.5** | HIV disease resulting in other mycoses |
| **B20.6** | HIV disease resulting in Pneumocystis jirovecii pneumonia |
| **B20.7** | HIV disease resulting in multiple infections |
| **B20.8** | HIV disease resulting in other infectious and parasitic diseases |
| **B20.9** | HIV disease resulting in unspecified infectious or parasitic disease |
| **B21** | Human immunodeficiency virus [HIV] disease resulting in malignant neoplasms |
| **B21.0** | HIV disease resulting in Kaposi sarcoma |
| **B21.1** | HIV disease resulting in Burkitt lymphoma |
| **B21.2** | HIV disease resulting in other types of non-Hodgkin lymphoma |
| **B21.3** | HIV disease resulting in other malignant neoplasms of lymphoid, haematopoietic and related tissue |
| **B21.7** | HIV disease resulting in multiple malignant neoplasms |
| **B21.8** | HIV disease resulting in other malignant neoplasms |
| **B21.9** | HIV disease resulting in unspecified malignant neoplasm |
| **B22** | Human immunodeficiency virus [HIV] disease resulting in other specified diseases |
| **B22.0** | HIV disease resulting in encephalopathy |
| **B22.1** | HIV disease resulting in lymphoid interstitial pneumonitis |
| **B22.2** | HIV disease resulting in wasting syndrome |
| **B22.7** | HIV disease resulting in multiple diseases classified elsewhere |
| **B23** | Human immunodeficiency virus [HIV] disease resulting in other conditions |
| **B23.0** | Acute HIV infection syndrome |
| **B23.1** | HIV disease resulting in (persistent) generalized lymphadenopathy |
| **B23.2** | HIV disease resulting in haematological and immunological abnormalities, not elsewhere classified |
| **B23.8** | HIV disease resulting in other specified conditions |
| **B24** | Unspecified human immunodeficiency virus [HIV] disease |
| **Z21** | Asymptomatic human immunodeficiency virus [HIV] infection status |
| **O98.7** | Human immunodeficiency [HIV] disease complicating pregnancy, childbirth and the puerperium |
| **R75** | Laboratory evidence of human immunodeficiency virus [HIV] |
| **D80** | Immunodeficiency with predominantly antibody defects |
| **D80.0** | Hereditary hypogammaglobulinaemia |
| **D80.1** | Nonfamilial hypogammaglobulinaemia |
| **D80.2** | Selective deficiency of immunoglobulin A [IgA] |
| **D80.3** | Selective deficiency of immunoglobulin G [IgG] subclasses |
| **D80.4** | Selective deficiency of immunoglobulin M [IgM] |
| **D80.5** | Immunodeficiency with increased immunoglobulin M [IgM] |
| **D80.6** | Antibody deficiency with near-normal immunoglobulins or with hyperimmunoglobulinaemia |
| **D80.7** | Transient hypogammaglobulinaemia of infancy |
| **D80.8** | Other immunodeficiencies with predominantly antibody defects |
| **D80.9** | Immunodeficiency with predominantly antibody defects, unspecified |
| **D81** | Combined immunodeficiencies |
| **D81.0** | Severe combined immunodeficiency [SCID] with reticular dysgenesis |
| **D81.1** | Severe combined immunodeficiency [SCID] with low T- and B-cell numbers |
| **D81.2** | Severe combined immunodeficiency [SCID] with low or normal B-cell numbers |
| **D81.3** | Adenosine deaminase [ADA] deficiency |
| **D81.4** | Nezelof syndrome |
| **D81.5** | Purine nucleoside phosphorylase [PNP] deficiency |
| **D81.6** | Major histocompatibility complex class I deficiency |
| **D81.7** | Major histocompatibility complex class II deficiency |
| **D81.8** | Other combined immunodeficiencies |
| **D81.9** | Combined immunodeficiency, unspecified |
| **D82** | Immunodeficiency associated with other major defects |
| **D82.0** | Wiskott-Aldrich syndrome |
| **D82.1** | Di George syndrome |
| **D82.2** | Immunodeficiency with short-limbed stature |
| **D82.3** | Immunodeficiency following hereditary defective response to Epstein-Barr virus |
| **D82.4** | Hyperimmunoglobulin E [IgE] syndrome |
| **D82.8** | Immunodeficiency associated with other specified major defects |
| **D82.9** | Immunodeficiency associated with major defect, unspecified |
| **D83** | Common variable immunodeficiency |
| **D83.0** | Common variable immunodeficiency with predominant abnormalities of B-cell numbers and function |
| **D83.1** | Common variable immunodeficiency with predominant immunoregulatory T-cell disorders |
| **D83.2** | Common variable immunodeficiency with autoantibodies to B- or T-cells |
| **D83.8** | Other common variable immunodeficiencies |
| **D83.9** | Common variable immunodeficiency, unspecified |
| **D84** | Other immunodeficiencies |
| **D84.0** | Lymphocyte function antigen-1 [LFA-1] defect |
| **D84.1** | Defects in the complement system |
| **D84.8** | Other specified immunodeficiencies |
| **D84.9** | Immunodeficiency, unspecified |
| **G11.3** | Cerebellar ataxia with defective DNA repair |
| **F02.4** | *Dementia in human immunodeficiency virus [HIV] disease (B22.0+) |
| **T86.1** | Kidney transplant failure and rejection |
| **T86.2** | Heart transplant failure and rejection |
| **T86.3** | Heart-lung transplant failure and rejection |
| **T86.4** | Liver transplant failure and rejection |
| **Z94.0** | Kidney transplant status |
| **Z94.1** | Heart transplant status |
| **Z94.2** | Lung transplant status |
| **Z94.3** | Heart and lungs transplant status |
| **Z94.4** | Liver transplant status |
| **Y83.0** | Surgical operation with transplant of whole organ |

**Supplementary Table S6:** Age of first reported allergic disease diagnosis in cases vs controls

| **Allergic Disease** | **Cases**  **(%)** | **Controls**  **(%)** | **P Value* ¥** |
| --- | --- | --- | --- |
| Asthma |  |  |  |
| Infant onset | 6 (2.6%) | 48 (4.3%) | 0.47* |
| Child onset | 142 (60.9%) | 688 (60.9%) |  |
| Adult onset | 85 (36.5%) | 393 (34.8%) |  |
| Median age of onset (IQR) | 11.6 years (5.5 – 24.5) | 12.3 years (5.4 – 23.5) | **0.73^¥^** |
| Eczema |  |  |  |
| Infant onset | 29 (11.2%) | 111 (9.6%) | 0.006* |
| Child onset | 92 (35.4%) | 535 (46.4%) |  |
| Adult onset | 139 (53.5%) | 508 (44.0%) |  |
| Median age of onset (IQR) | 19.8 years (6.5 – 32.7) | 14.9 years (4.0 – 27.7) | **0.004^¥^** |
| Allergic Rhinitis |  |  |  |
| Infant onset | 0 (0.0%) | 4 (0.4%) | 0.24* |
| Child onset | 94 (54.7%) | 445 (48.5%) |  |
| Adult onset | 78 (45.4%) | 469 (51.1%) |  |
| Median age of onset (IQR) | 17.0 years (10.7 – 28.0) | 18.4 years (11.5 – 28.4) | **0.40^¥^** |

*P value from Chi-squared test; **^¥^** p value from Mann-Whitney U test

**Supplementary Table S7:** Stratum-specific Odds Ratios for the effect of eczema on HL risk by age of first reported eczema

| **Age of first eczema reporting** | **Odds ratio (OR)**  **95%CI** | **P Value** | **Adjusted OR**  **95%CI** | **P value** | **Adjusted OR**  **After adjustment for steroids** | **P value** |
| --- | --- | --- | --- | --- | --- | --- |
| Infant onset | 1.68 (1.09 – 2.58) | 0.019 | 1.53 (0.99 – 2.36) | 0.058 | 1.43 (0.92 – 2.21) | 0.11 |
| Child onset | 1.10 (0.86 – 1.40) | 0.47 | 1.05 (0.82 – 1.34) | 0.70 | 0.96 (0.74 – 1.23) | 0.73 |
| Adult onset | 1.80 (1.46 – 2.21) | <0.001 | 1.73 (1.40 – 2.13) | <0.001 | 1.54 (1.24 – 1.90) | <0.001 |

**Supplementary Table S8:** Summary of studies investigating associations between allergic disease and Hodgkin’s lymphoma. OR, odds ratio; CI, confidence interval

| **Study, Year** | **Study Design** | **Study Size** | **Population** | **Data Source** | **Outcomes (OR, 95%CI)** | **Comments** |
| --- | --- | --- | --- | --- | --- | --- |
| Söderberg et al, 2006 | Population based case-control study | 2394 Cases  149,344 Controls | Sweden, ages 0-110, 1987-1999. Cases: cancer registry; Controls: random population sample | Swedish Hospital Discharge Registry | Asthma  (0.6, 0.4-0.9) | OR based on 18 exposed cases |
| Vineis et al, 2000 | Multicenter case-control study | 354 Cases  1718 Controls | Italy, ages 20-74, 1990-1993. Cases: periodic surveys of hospitals; Controls: random population sample | Face-to-face interviews | Hay fever  (0.5, 0.3-0.8)  Eczema  (0.7, 0.4-1.3) |  |
| Cozen et al, 2009 | Cohort, Twin-study | 188 YAHL discordant twin pairs | USA, Twins aged<50, born 1980-1992. Recruited from advertisements in print media | Questionnaire | Eczema  (4.2, 1.2-14.8) | Results based on 19 twin pairs discordant for the exposure |
| Hollander et al, 2015 | Population based case-control study | 585 Cases  3187 Controls | Sweden and Denmark, ages 18-74, 1999-2002. Cases: cancer registry; Controls: random population sample | Telephone interview | Asthma  (0.81, 0.54-1.20)  Eczema  (0.82, 0.56-1.20)  Hay fever  (0.81, 0.64-1.03) |  |
| Linabery et al, 2014 | Multicenter case-control study as part of Children’s Oncology Group (COG) study | 517 Cases  784 Controls | USA, Puerto Rico and Canada, ages 0-14, 1989-2003. Cases: COG registry; Controls: random population sample | Telephone interview | Asthma  (1.20, 0.81-1.77)  Eczema  (1.52, 0.89-2.62)  Hay fever  (0.88, 0.60-1.30) |  |
| Dikalioti et al, 2012 | Multicenter case-control study | 111 Cases  111 Controls | Greece, ages 0-14, 1996-2008. Cases: hospital registers from 6 centers; Controls: hospital based | Parent questionnaire | Asthma  (0.66, 0.28-1.57) |  |
| Becker et al, 2007 | Multicenter case-control study as part of the Epilymph study | 336 Cases  2427 Controls | 7 European countries, ages 0-71, 1998-2004. Cases: cancer/hospital registry; Controls: random population sample/hospital based | Questionnaire | Respiratory Allergy (hay fever or asthma)  (0.79, 0.56-1.12) |  |
